# Neural response to aggressive and positive interactions in violent offenders and nonviolent individuals

**DOI:** 10.1002/brb3.2400

**Published:** 2021-11-10

**Authors:** Svenja Taubner, Sophie Hauschild, David Wisniewski, Silke Wolter, Gerhard Roth, Thorsten Fehr

**Affiliations:** ^1^ Institute for Psychosocial Prevention University Hospital of the University Heidelberg Heidelberg Germany; ^2^ Psychological Institute Heidelberg University Heidelberg Germany; ^3^ Department of Experimental Psychology Ghent University Ghent Belgium; ^4^ Brain Research Institute University of Bremen Bermen Germany; ^5^ Center for Cognitive Sciences Department of Neuropsychology University of Bremen Bremen Germany; ^6^ Center for Advanced Imaging Universities of Bremen and Magdeburg Germany

**Keywords:** amygdala, fMRI, interpersonal provocation, peri‐aqueductal gray, quasi‐realistic experimental design, violence

## Abstract

**Background:**

Due to its severe negative consequences, human violence has been targeted by a vast number of studies. Yet, neurobiological mechanisms underlying violence are still widely unclear and it seems necessary to aim for high ecological validity to learn about mechanisms contributing to violence in real life.

**Methods:**

The present functional magnetic resonance imaging (fMRI) study investigated the neurofunction of individuals with a history of violent offenses compared with that of controls using a laboratory paradigm requesting individuals to empathically engage in videos depicting provocative aggressive and positive social interactions from a first‐person perspective.

**Results:**

The contrast of aggressive vs. positive scenarios revealed midbrain activation patterns associated with caudal periaqueductal gray (PAG) in violent offenders; In controls, the rostral PAG was involved. Additionally, only in controls, this contrast revealed an involvement of the amygdaloidal complex. Moreover, in violent offenders the contrast of positive vs. aggressive situations revealed an involvement of areas in the insula, post‐central gyrus and anterior cingulate cortex.

**Conclusions:**

Our results support findings on the differential role of PAG subdivisions in response to threat and point to altered processing of positive social interactions in violent offenders. They further support the notion that changes in PAG recruitment might contribute to violent individuals “taking action” instead of freezing in case of threatening situations.

## Abstract 2

Violent offenders show activation in the caudal peri‐aqueductal gray (PAG) when mentally engaging in aggressive versus positive video scenarios. Control individuals show activation in the rostral PAG during the respective contrast. Both subdivisions of the PAG have been associated with different responses to threat. Differences in PAG recruitment might contribute to violent individuals “taking action” instead of freezing in case of threatening situations.

## 1 INTRODUCTION

### 1.1 THE “VIOLENCE NETWORK(S)”

Human violence has severe negative consequences for micro‐, meso‐ and macrosocial systems. Thus, factors underlying the occurrence of violence have been and remain of great research interest. At a neurobiological level, there is increasing consensus about a network of brain regions functionally contributing to human violence (e.g., Fanning et al., 2017; Raine, 2019; Rosell & Siever, 2015). Networks implicated are the “neuro‐moral network” (Raine, 2019) and an amygdala‐frontal circuit (Rosell & Siever, 2015). These networks include cortical areas, subcortical limbic regions and areas of the midbrain.

Concerning the cortex, studies investigating individuals displaying aggressive behaviour as a trait (for review, see McKinley et al., 2018) or after a traumatic brain injury (Darby, 2017) suggest that deviations in frontal and temporal cortices underlie violence. According to the studies, aggressive behaviour can result from dysfunctional emotion regulation, impaired inhibition of action, difficulties with reaching appropriate moral judgments and with using them as a basis of (re)action (cf. Darby, 2017; McKinley et al., 2018; Raine, 2019). Within the subcortical parts of the limbic system, the amygdala has been linked to the occurrence of violence due to its role in processing emotionally relevant input, enabling emotional learning and “emotionally triggered” reactions via its interconnectedness with prefrontal and temporal areas as well as the midbrain (cf. Rosell & Siever, 2015). Of midbrain centers, the periaqueductal gray (PAG) has been included into the neurobiological circuitry underlying violence because of its role in freeze, flight and, importantly, fight reactions upon threat (Roelofs, 2017). In line with findings previously revealed by studies investigating the role of the PAG in animals (e.g., Depaulis et al., 1992), human fight and its reactions have been linked with the activation of the caudal, or dorsolateral, subdivision of the PAG (dlPAG; Roelofs, 2017), while activation of the rostral, or ventrolateral, subdivision of the PAG (vlPAG) is proposed to be related to inhibiting fight and flight reactions. Depending on input by the amygdala, activation in the vlPAG blocks fight/flight reactions and initiates freezing instead (Roelofs, 2017).

### 1.2 Different functional mechanisms within the “violence network”?

Research leading to the proposition of a neurobiological network underlying violence has largely been derived from the description of two clinically relevant phenotypes of individuals exhibiting violence: Individuals from the first phenotype are described to act violently without an observable trigger to achieve certain goals, that is, *proactively* or *instrumentally*. Individuals of the second phenotype show violence mostly following a trigger (e.g., provocation or frustration), that is, they exhibit violence reactively or impulsively. This phenotype has been associated with a hostile attribution bias, that is, the tendency of individuals to over‐attribute hostile intentions in others even in the face of nonhostile social cues (cf., e.g., Card & Little, 2006). Different functional mechanisms of amygdala and frontotemporal activation have been associated with the two phenotypes: A hypoactivation of the amygdala (Lozier et al., 2014 in Rosell & Siever, 2015) in combination with a “neuromoral” dysfunction, that is, impairment in the functioning of prefrontal areas implicated in moral decision making (e.g., OFC), is assumed to underlie *proactive* aggression (Raine, 2019). In contrast, a hyperactivation of the amygdala in response to a trigger in combination with a failure to prefrontally downregulate this heightened activation constitutes an “emotional hyperreactivity” and underlies reactive violence (Rosell & Siever, 2015).

Scientifically relevant and clinically useful as this distinction has been, authors argue for “differences in degree rather than in kind” (Raine, 2019). Indeed, we are confronted with the reality that most violent individuals cannot easily be assigned to one group or the other (Raine, 2019; Rosell & Siever, 2015). Moreover, based on the assumption of a neurobiological network contributing to violence, we must conclude that the occurrence of violence might be a result of impairment in any of the implicated network regions or dysfunctions in their connectivity, leaving the neurobiological characteristics underlying violence still widely unclear.

### 1.3 Social context of violence and the present study

In addition to individual characteristics, the situational context is an important aspect of violent impulses actually being put into action. Specifically, interpersonal provocation and/or proximal threat is considered to be one main reason for human aggression (cf. Anderson & Bushman, 2002; Fehr & Achtziger, 2021; Fehr et al., 2014). Widely established laboratory measures of aggression using noise blasts or money subtraction have been discussed to provoke a revenge‐like, minor reactive aggression in the sense of tit for tat (Fehr et al., 2014; Ferguson & Dyck, 2012). However, they might not provoke a *defence‐like* reactive aggression as a response to interpersonal provocation or proximal threat. As such, their expressive power regarding real‐life, potentially maladaptive forms aggression might be limited (cf. Chester & Lasko, 2018; Fehr & Achtziger, 2021; Fehr et al., 2014; Ferguson & Dyck, 2012). Thus, in order to improve ecological validity, it seems relevant to (1) investigate individuals who actually have committed real violent offenses and (2) aim for experimental approaches simulating a context in which violence frequently occurs in a quasi‐realistic way. For this purpose, we investigated brain physiological correlates during the presentation of provocative aggressive in contrast to social positive video scenarios in a group of individuals who committed violent offenses and in a group of nonviolent individuals.

Based on the data presented by Fehr et al. (2014), we expected for all participants confronted with provocative aggressive versus social positive scenarios that at iso‐cortical level distributed activation patterns occur in several heteromodal association cortices located in the superior parietal, lateral parieto‐temporal, lateral occipito‐temporal and prefrontal (premotor) brain areas. These were discussed to be associated with a prototypic and lifelong learned perception‐action‐cycle brain network related to reactive aggressive behaviours as response to (here, quasi‐realistic) proximal threat displayed in the video scenarios used as stimulation (see also the concept of the emotional body language network proposed by De Gelder et al. 2006). Additionally, we expected right inferior frontal brain activation patterns, which have been discussed as an inhibiting instance, as the participants were indeed asked to show no real motor behaviour in the scanner tube, or because they would suppress reactive aggressive motor behaviour driven by concepts of adequate conflict reducing control (cf. Fehr et al., 2014).

We expected specific patterns of activation for violent offenders and nonviolent controls when confronted with the provocative aggressive versus social positive scenarios in regions previously implicated in violence, including the amygdaloid complex and midbrain structures.

As individuals executing violence often have a history of experiencing violence themselves throughout their lives (e.g., Afifi et al., 2019), we assumed that comparing violent offenders’ neurofunction during aggressive versus positive social scenarios might reveal different activation patterns in experience‐related perception‐action cycle systems than the respective contrast in CON individuals.

The amygdaloid complex (AMY) and associated neural networks have been viewed to optionally provide an important instance in both conscious and automatic, preattentive evaluation of emotional context aspects and have especially been implicated in the processing of fear (e.g., LeDoux & Phelps, 2008; Pehlps & LeDoux, 2005). Furthermore, a downregulation or lack of involvement of the amygdaloid complex in emotional context was discussed for psychopathic individuals and/or chronic violent offenders (e.g., Raine, 2019). Thus, we also expected contrasts to reveal specific patterns of activation in the amygdaloid complex for violent offenders and control participants.

The midbrain structure PAG was discussed as one of the most crucial brain areas to be involved in proximal threat‐induced flight‐ and fight‐behaviours in both animals (e.g., Depaulis et al., 1992) and humans (e.g., Roelofs, 2017). Based on findings by Fehr et al. (2014), we likewise expected midbrain structures, such as the PAG, to be particularly involved in the processing of provocative aggressive scenarios with quasi‐realistic proximal threat character when contrasted to social positive scenarios. Respective regional activation patterns were explored for violent offenders and the control group separately. As working hypotheses based on the data and models provided by Depaulis et al. (1992) and Roelofs (2017), we expected rather caudal, or to say dorsal, midbrain (PAG) activation patterns in violent offenders (facilitating attack behaviours), and rostral, or to say ventral, activation patterns in this region in controls (i.e., inhibiting attack behaviours).

## 2 EXPERIMENTAL PROCEDURE

### 2.1 Study participants

Participants were recruited via social media and social services and included if they were male and between 17 and 24 years old. The sample consisted of two sub‐samples: The group of male violent offenders (“violent group,” VIOL; *n*_1 _= 25; mean age 19.9, SD = 1.7) was recruited from probation services, victim‐offender mediation and participants in a court‐ordered pedagogic training upon being convicted of a criminal offense. The control group without a history of violent offenses was matched for age (CON; *n*_2 _= 21; AM = 20.0, SD = 1.3). Participants were informed that the aims of the study are a better understanding of aggression and to increase knowledge about the effectivity of pedagogic measures. In the VIOL group, 22 fulfilled criteria of antisocial personality disorder and 21 of conduct disorder according to the Structured Clinical Interview for DSM‐5 (SCID II; First, 2015). Two participants of the VIOL group were accommodated in a correctional facility. CON participants did not report any history of psychiatric or neurological illness. None of the participants reported abnormal vision, regular drug use or current medication with psychotropic side effects. All participants were right‐handed according to the Edinburgh Handedness Inventory (Oldfield, 1971), and there was no contraindication for an fMRI investigation. Participants were excluded if their IQ measured with the Culture Fair Test (Weiß, 2006) was lower than 80. Mean IQ in the VIOL group (AM = 108; SD = 15.5) differed significantly from the CON group (AM = 118.2; *SD *= 14.8; *t *= −2.1, *p *= .037; see Table 1 and Figure 2c, right panel). Years of education were available for 20 VIOL (AM = 11.3, SD = 1.5) and 16 CON participants (AM = 12.7, SD = 1.0); the mean was lower in the VIOL group (*t *= −3.2, *p *= .003). All participants were familiarized with the stimulus presentation, informed about the procedure and finally gave a written and informed consent to participate. The experimental set‐up was designed according to the Declaration of Helsinki of the World Medical Association, and the study protocol was approved by the local ethics committee of the University Hospital Charité Berlin. All participants were paid €50 after participation.

**TABLE 1:** Sample characteristics

	VIOL (N = 25)		CON (N = 21)			
	AM/freq	SD	AM	SD	*t*	*p*
Age	19.9	1.7	20.0	1.3	−.2	.876
CFT	108.6	15.5	118.2	14.4	−2.2	.037
RPQ total	18.1	5.5	10.7	5.4	4.6	<.001
RPQ reactive	12.3	3.1	7.9	3.1	4.7	<.001
RPQ proactive	5.8	3.2	2.8	2.9	3.4	.001
ASPD	22	–	–	–	–	–
CD	21	–	–	–	–	–

*Note*: Missing CFT score and age of one control participant were substituted by the mean of the CON group.

Abbreviation: RPQ, Reactive‐Proactive Aggression Questionnaire

### 2.2 Procedure

**FIGURE 1:**
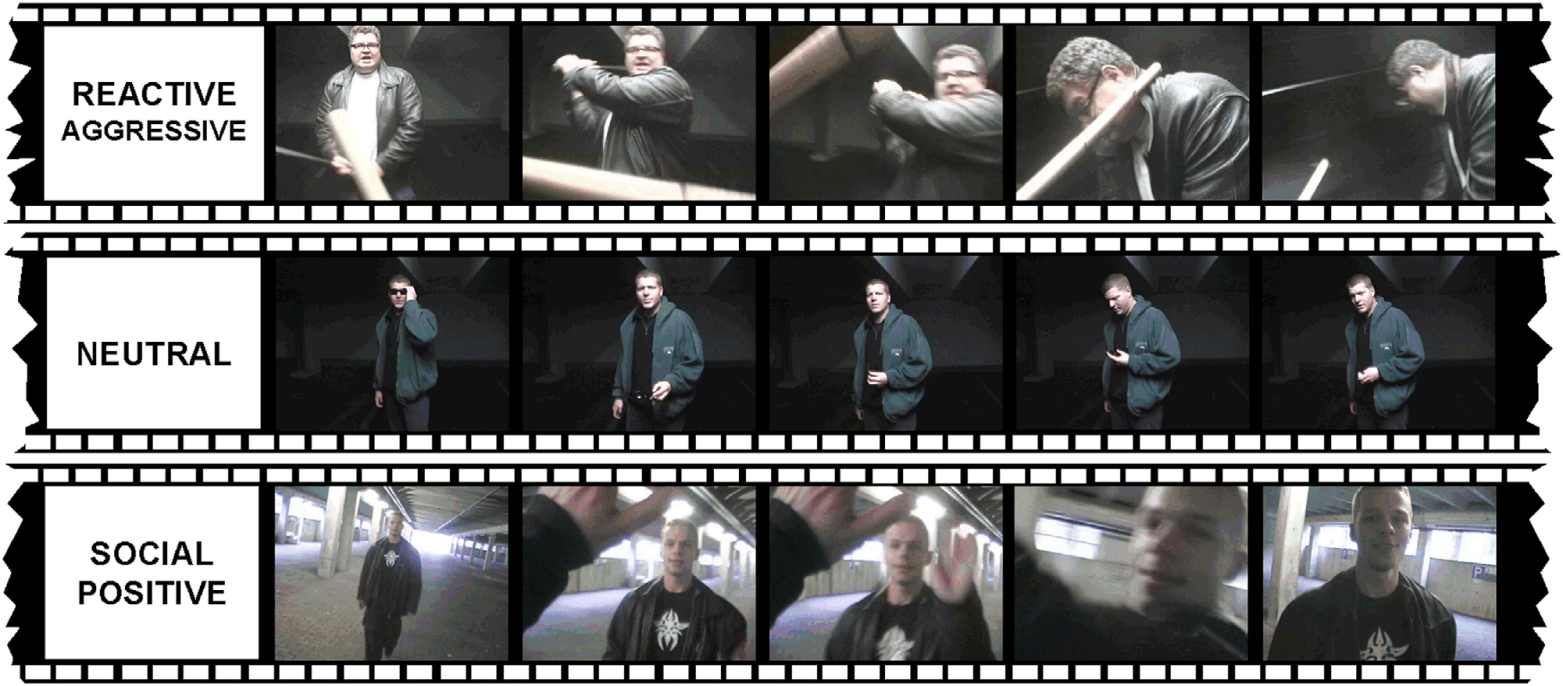
Illustration of reactive aggressive, neutral and social positive interaction video stimuli (captured frames of the respective types of scenarios)

Participants were asked to attentively watch and empathically engage in 90 different video‐scenarios (of 5–6 s length; visual angle below 4.0°; presented twice in random order across six experimental runs making up 180 trials at all). Videos were taken from an inventory (Fehr et al., 2014), which had been especially developed and evaluated to investigate neural processes involved in the processing of human anger, violence (in the present selection of stimuli with reactive aggressive scenarios) but also social positive interaction. Neutral (N: nonaffective interaction), social positive (P: scenarios such as hand‐shaking) and aggressive violence‐related (A: being pushed and push the assailant back, that is, reactive aggression in response to a provocation) video‐scenarios (30 different scenarios from each category presented twice in the experiment) showing complex social interactions from a first‐person perspective were presented (see Figure 1 for trial design). This kind of personal objective view facilitates participants to feel quasi‐realistically to be part of the displayed interaction. The stimuli provide a proximal stimulation triggering different emotional responses as evaluated by Fehr et al. (2014). Between the presentation of each video‐clip, a fixation cross was presented between 4 and 8 s. After the fMRI experiment, participants watched all video‐scenarios again (outside the scanner). They were asked to rate INTENSITY and VALENCE on a five‐point scale each according to Bradley and Lang (1994). Moreover, participants’ aggression was assessed via the Reactive‐Proactive Aggression Questionnaire (RPQ; Raine et al., 2006).

### 2.3 Behavioural data analyses

Video scenarios were rated after the scanner session according to two scales introduced by Bradley and Lang (1994), INTENSITY (10 leveled scale from lowest intensity 0 to highest intensity 9), and VALENCE (11 leveled scale from negative −5 to positive 5). Subsequent post hoc tests between participants’ ratings on the scales were calculated by means of paired *T*‐tests in case of within‐group comparisons between stimulus conditions and by means of *T*‐tests for independent samples in case of between‐group comparisons. All findings of the analyses of variance (ANOVAs) were Greenhouse–Geisser (GG) adjusted.

### 2.4 Imaging data acquisition and analyses

#### 2.4.1 Data acquisition, preprocessing and regressor‐configuration

The functional and structural MRI data were obtained on a 3T Siemens Trio scanner equipped with a 12‐channel head coil. Changes in blood oxygenation level‐dependent (BOLD) T2*‐weighted MR signal were measured using a gradient echo‐planar imaging (EPI) sequence (33 axial [AC‐PC] slices in descending acquisition order with whole brain coverage; FOV = 192 mm, 64 × 64 matrix; TR = 2000 ms; TE = 30 ms; flip angle = 78°; voxel size 3 × 3 × 3.75 mm^3^). Note that 1050 volumes were obtained during six runs (175 volumes each run) during 35 min measurement time. Structural MRI data were collected after the functional scanning runs (MPRAGE [magnetization prepared rage]); 192 slices, slice thickness of 1 mm, FOV = 256 × 256; matrix: 256 × 256, TR = 1900 ms, TE = 2.52 ms; resulting in 1 mm^3^ voxel size).

Image analysis was performed using the Statistical Parametric Mapping software package (SPM8, Welcome Department of Cognitive Neurology, London, UK) on a Matlab R2008a platform (The MathWorks, Natick, MA). For each session and participant, images were slice‐time corrected, motion‐estimated, realigned, normalized to the Montreal Neurological Institute Template (MNI; Collins, 1994) and smoothed using an isotropic Gaussian kernel (full width half maximum = 8 mm) prior to further analysis. Global effects (grand mean scaling over all volumes) were removed from the functional MRI data, and a high‐pass filter (128 s) was applied to remove low‐frequency signal drifts.

Design matrices for GLM processing included four regressors in all: one regressor for each of the three 5 s taking emotional scenario categories provocative aggressive, social positive and neutral scenarios, and one more for the between‐trial fixation cross displayed between 4 and 8 s. All trial events were modeled by the standard hemodynamic response function.

#### 2.4.2 Contrasting and statistical processing of functional imaging data

The preprocessed data sets were analyzed by calculating a *t*‐statistic for different contrasts between stimulus categories. In the present study, contrasts between reactive aggressive (A) and social positive (P) scenario‐types were reported. Second‐level random effects analyses (Holmes & Friston, 1998) were performed on individual contrast images to identify the main task effects by means of a one‐sample *T*‐test. A statistical threshold of *p* < .001 (corrected by an ad hoc determined lowered significance threshold, *k* ≥ 10 voxel cluster size) was applied to identify significant activation clusters. Statistical MNI‐coordinates of peak activations were converted from the SPM8‐output into Talairach space with a transformation algorithm, and a reference template based on the Talairach‐atlas (Talairach & Tournoux, 1988) was used to determine the respective anatomical regions. Common activation patterns for A versus P scenario types were investigated by conjunction analyses (*p* < .05; FDR‐corrected, *k* ≥ 5) (see Friston et al., 2005). All above‐listed statistical analyses were separately performed for the VIOL and CON groups. For a detailed comparison between second‐level random effects analyses with and without CFT‐values as covariate, see Tables S3.1–S3.4 in the Supporting Information Appendix).

To inspire future research, exploratory correlations between contrast images reactive aggressive versus social positive (a > p) video scenarios and both RPQ total values (see Supporting Information Appendix 1 ) and valence ratings (see Supporting Information Appendix 2) were calculated for selected regions of interest (ROIs), which is related to emotional stimulus processing (i.e., areas in the limbic system and brainstem).

## 3 RESULTS

### 3.1 Self‐reported aggression

**FIGURE 2:**
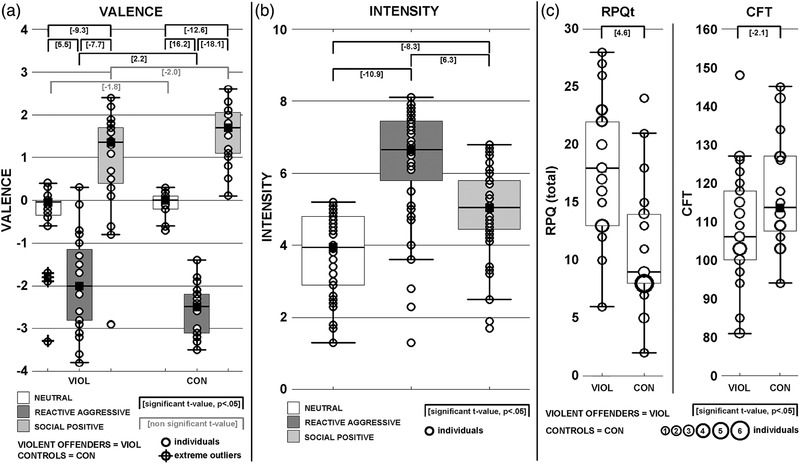
Post hoc evaluation of the video‐stimuli concerning (a) VALENCE and (b) INTENSITY according to Bradley and Lang (1994); furthermore, group‐differences for Reactive‐Proactive Aggression Questionnaire (RPQ) (c, left panel) and CFT (c, right panel) values were illustrated

*T*‐tests for independent samples revealed significantly higher self‐reported overall, reactive and proactive aggression measured with the RPQ in VIOL compared to CON participants (all *p* < .002, see Table 1 and Figure 2c, left panel).

### 3.2 Behavioural data

There were no group differences in INTENSITY ratings. However, interaction analyses on INTENSITY ratings as performed by repeated measurement ANOVAs including the dependent factor Experimental Condition (EC: three levels comprising N, P and A), and the independent factor GROUP (two levels) revealed a main effect for EC (*F*_(2,84) _= 84.2; *p* < .01, GG‐Epsilon = .8). Video‐scenarios showing neutral interactions (N‐scenarios) were rated the lowest (3.8 ± 1.1) and video‐scenarios showing reactive–aggressive interactions (A‐scenarios) were rated the highest (6.7 ± 1.1) in INTENSITY. Social positive video‐scenarios (P‐scenarios) ranged in‐between (5.0 ± 1.2) the A‐and the N‐scenarios (all post hoc comparisons *p* < .01 for pooled VIOL and CON participants; for detailed illustration and respective *t*‐values, see Figure 2b).

There were group‐specific differences in EC‐related VALENCE ratings (for detailed illustration and respective *t*‐values, see Figure 2a). Interaction analyses on VALENCE‐ratings as performed by a repeated measurement ANOVA including the dependent factor EC (three levels comprising N, P and A) and the independent factor GROUP (two levels) revealed an interaction effect EC x group (*F*_(2,84) _= 6.1; *p* < .01, GG‐Epsilon = .6) and a main effect for EC (*F*_(2,84) _= 199.9; *p* < .01, GG‐Epsilon = .6). In both groups, N‐scenarios (VIOL: −0.4 ± 0.9; CON: −0.1 ± 0.3) produced higher ratings than A‐scenarios (VIOL: −2.0 ± 1.1, *t* = 5.5, CON: −2.6 ± 0.6; *t* = 16.2) and lower values than P‐scenarios (VIOL: −0.4 ± 0.9, *t* = −9.3; CON: −0.1 ± 0.3, *t* = −12.6) (all dependent *T*‐tests *p* < .01). In both groups, A‐scenarios were rated significantly negative and P‐scenarios were rated significantly positive, whereas N‐scenarios did not differ significantly from zero (all tests against zero *p* < .01). Groups differed in A‐scenarios such as CON showed more negative ratings compared to VIOL participants (*p* < .01, *t* = 2.2). In N‐scenarios, there were pronounced, but nonsignificant, more negative ratings in VIOL participants (*p* = .087, *t* = −1.8), and in P‐scenarios there were nonsignificant, higher positive ratings in CON (*p* = .05, *t* = −2.0). Groups showed in all EC heteroscedasticity with higher standard deviations in VIOL individuals as compared to CON (all *F‐*tests *p* < .01).

### 3.3 FMRI data

#### 3.3.1 Reactive–aggressive (A) versus social–positive (P) interaction scenarios

**FIGURE 3:**
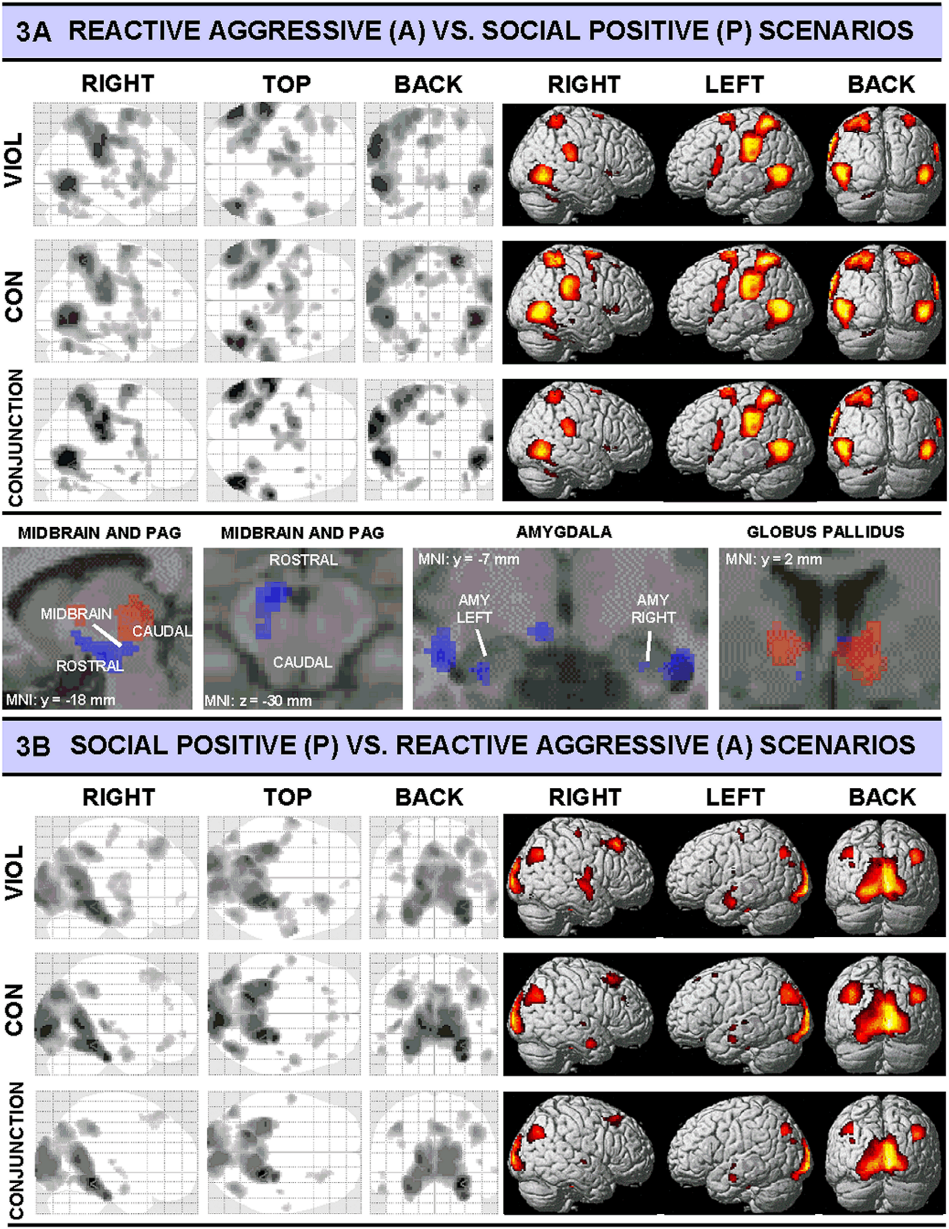
Glass‐brain views and rendered statistics on standard MNI‐brain template for the contrasts reactive aggressive versus social positive interaction scenarios (upper panel) and vice versa (lower panel) for each group (violent individuals = VIOL; controls = CON) and the respective conjunction (null) analyses over groups; lower part of the upper panel shows selected section views for four regions of interest (VIOL = red shadings, CON = blue shadings). Involved regions for each group and conjunction analyses are listed in Table 2. Anatomical regions, detailed peak activation *t*‐values and Talairach‐coordinates are listed in Tables S3 and S 4 in the supporting document. All contrasts were *p* < .001, uncorrected, with a minimum cluster size of *k* = 10 voxels

At an iso‐cortical level, in both groups the contrast A versus P revealed distributed activation patterns in superior parieto‐temporal, occipito‐temporal, precentral and premotor areas (for illustration, see Figure 3a upper left and right panels, for an overview of recruited brain regions see Table 2, upper part, and for a detailed list of activation foci, coordinates and *t*‐values, see Table 3). At the sub‐cortical level, both groups showed partially overlapping (conjunct) and partially distinct distributed activation patterns in the cingulate gyrus, insula and midbrain (VIOL individuals caudal and dorsal and CON rather rostral and ventral) in the anterior PAG (see Figure 3a, lower panel, left two section views for detailed illustration) regions. Furthermore, both groups showed the involvement of cerebellar brain areas. VIOL individuals specifically recruited parts of the globus pallidus (see Figure 3a, lower panel, section view for detailed illustration), and CON specifically recruited areas in and adjacent to the amygdaloidal complex (i.e., parahippocampal gyrus and uncus, see Figure 3a, lower panel, middle right section view for detailed illustration).

**TABLE 2:** Summary of fMRI results during the contrasts A versus P and P versus A

	Reactive aggression (A) versus social positive (P)			
Anatomical structure involved	VIOL	CON	CONJ	Explanation
Precentral gyrus	L	R	–	VIOL left‐ and CON right‐hemispheric
Superior frontal gyrus	L/R	L/R	L/R	Both groups bi‐lateral with overlap
Medial frontal gyrus	L	R	–	VIOL left‐ and con right‐hemispheric
Inferior frontal gyrus	L/R	L/R	L/R	Both groups bi‐lateral with overlap
Between insula, IFG and precentral gyrus	–	L	–	Only CON left‐lateral
Cingulate gyrus	L	L	L	Both groups with overlap
Insula	L	L	–	Both groups left‐lateral; different sub‐regions
Postcentral gyrus	L/R	L/R	L/R	Both groups bi‐lateral with overlap
Between postcentral G. and paracentral L.	–	R	–	Only CON right lateral
Superior parietal lobule	L/R	L/R	L/R	Both groups bi‐lateral with overlap
Inferior parietal lobule	L	L/R	L	VIOL only left‐lateral with overlap
Precuneus	–	L	–	Only CON left‐lateral
Fusiform gyrus	R	L/R	L/R	Both groups "bi‐lateral" with overlap ^*)^
Middle occipital gyrus	–	L	–	Only CON left‐lateral
Superior temporal gyrus	R	R	L/R	Both groups "right‐lateral" with overlap ^*)^
Middle temporal gyrus	L	L/R	L	VIOL only left‐lateral with overlap
Inferior temporal gyrus	L/R	R	L/R	Both groups "bi‐lateral" with overlap ^*)^
Parahippocampal gyrus/amygdala	–	R	–	Only CON right‐lateral
Uncus/amygdala	–	L	–	Only CON left‐lateral
Uncus	–	L	–	Only CON left‐lateral
Between caudate/caudate tail and PHG	L	–	L	Both groups "left‐lateral" with overlap ^*)^
Brainstem/midbrain	L	L/R	–	VIOL only left‐lateral; different sub‐regions
Brainstem/pons	–	R	–	Only CON right‐lateral
Lentiform nucleus/medial globus pallidus	L	–	–	Only VIOL left‐lateral
Lentiform nucleus/lateral globus pallidus	R	–	–	Only VIOL right‐lateral
Thalamus	L	–	–	Only VIOL left‐lateral
Thalamus/pulvinar	–	L	–	Only CON left‐lateral
Between thalamus and caudate head	–	R	R	Both groups "right‐lateral" with overlap ^*)^
Cerebellum/anterior lobe/culmen	L/R	L/R	R	Both groups bi‐lateral with overlap right‐lateral
Cerebellum/posterior lobe/declive	L/R	L	L/R	Both groups "bi‐lateral" with overlap ^*)^
Cerebellum/posterior lobe/tuber	R	–	–	Only VIOL right‐lateral
Cerebellum/posterior lobe/uvula	–	–	L	Both groups overlap "left‐lateral" ^*)^

	Social positive (P) versus reactive aggression (A)			
Anatomical structure involved	VIOL	CON	CONJ	Explanation
Precentral gyrus	L/R	–	–	Only VIOL bi‐lateral
Superior frontal gyrus	R	L/R	R	VIOL only right‐lateral with overlap
Medial frontal gyrus	R	L/R	–	VIOL only right; different sub‐regions
Middle frontal gyrus	L/R	L/R	R	Both groups bi‐lateral; only right‐lat. overlap
Anterior cingulate	R	–	–	Only VIOL right‐lateral
Cingulate gyrus	R	R	R	Both groups right‐lateral with overlap
Posterior cingulate	L/R	L/R	L/R	Both groups bi‐lateral with overlap
Insula	L/R	–	–	Only VIOL bi‐lateral
Postcentral gyrus	R	–	–	Only VIOL right‐lateral
Inferior parietal lobule	R	–	R	Both groups "right‐lateral" with overlap ^*)^
Precuneus	L	L/R	L	VIOL only left‐lateral with overlap
Superior occipital gyrus	L	–	L	Both groups "left‐lateral" with overlap ^*)^
Cuneus	L	L	L/R	Both groups left‐lateral with "bi‐lat." overlap ^*)^
Lingual gyrus	L	R	–	Only VIOL left‐lateral and CON right‐lateral
Fusiform gyrus	L	L	L	Both groups left‐lateral with overlap
Superior temporal gyrus	L/R	L/R	L/R	Both groups bi‐lateral with overlap
Middle temporal gyrus	L/R	L	–	CON only left‐lateral; different sub‐regions
Inferior temporal gyrus	L	L/R	L	CON only left‐lateral with overlap
Parahippocampal gyrus	L/R	L/R	L/R	Both groups bi‐lateral with overlap

*Note*. List of brain regions showing at least one focus of activation for the contrast A versus P (upper panel) and P versus A (lower panel), separately for violent individuals (VIOL), controls (CON) and the conjunction (CONJ) between both groups. L means left hemispheric and R means right hemispheric foci in the respective anatomical regions.

*) = Within‐group‐ and conjunction analyses produce an inconsistent picture due to the different underlying statistical complexities of the applied algorithms (*t*‐test vs. conjunction analyses). Detailed information about coordinates and *t*‐values are listed in an online supporting document in Tables S1 and S2.

**TABLE 3:** Detailed fMRI data for contrast A versus P

BOLD contrast: Reactive aggressive versus social positive condition (a > p)													
	VIOL					CON				VIOL and CON			
Anatomical region	H	t	x	y	z	t	x	y	z	t	x	y	z
Precentral gyrus	L	5.1	−57	12	5								
	R					4.0	48	2	37				
	R					3.5	40	1	26				
Superior frontal gyrus	L	6.7	−16	1	63	6.6	−10	−3	66	6.2	−14	1	64
	L					6.0	−22	−5	63				
	L					4.3	−42	−4	46				
	R	5.2	10	9	62	5.1	10	1	68	4.5	10	7	64
	R	4.7	6	18	58	5.0	18	1	64	4.1	2	0	68
	R	4.7	2	3	66	4.3	8	28	50				
	R					3.4	12	20	51				
Medial frontal gyrus	L					3.9	−8	46	22				
	L	4.9	−24	−7	57					4.9	−24	−7	57
	L	4.7	−40	40	22								
	R					4.3	26	−5	59				
	R					4.7	38	−1	50				
Inferior frontal gyrus	L	5.8	−51	8	14	6.1	−53	7	29	5.3	−51	9	16
	L	4.7	−38	15	−14	5.4	−53	8	14	4.5	−53	9	29
	L	4.6	−44	17	−1	4.6	−30	17	−14	3.8	−36	17	−11
	L	4.5	−53	9	29								
	L	4.6	−44	35	6								
	R	5.5	55	19	−1	4.3	42	25	2	3.6	51	17	−3
	R	4.7	53	31	−7	3.6	51	17	−3	3.5	44	25	−1
Between insula, IFG and precentral gyrus	R					3.5	51	12	3				
Cingulate gyrus	L	6.5	−6	−17	40	6.8	−12	−25	40	5.5	−8	−21	40
	L	3.8	−4	13	27	4.7	−2	−2	33	4.7	−2	−4	33
	R	4.8	8	−17	41	4.6	10	−19	40	4.4	10	−17	40
Insula	L	4.6	−36	20	3	4.0	−48	−36	20				
Postcentral gyrus	L	8.0	−55	−25	40	7.3	−65	−24	21	7.0	−53	−25	40
	L	6.8	−53	−29	51	7.0	−53	−25	40				
	L					6.6	−61	−22	32				
	R	6.7	63	−26	20	7.2	65	−28	20	6.7	63	−26	20
	R	4.3	34	−41	68								
	R	3.6	34	−32	62								
Between postcentral G. and paracentral L.	R					3.9	10	−39	68				
Superior parietal lobule	L	6.8	−24	−48	59	6.7	−24	−51	60	6.5	−24	−49	60
	R	5.9	32	−46	58	10.1	30	−46	58	5.9	32	−46	58
Inferior parietal lobule	L	8.4	−59	−33	31	7.3	−57	−24	25	6.9	−61	−28	27
	L	6.3	−36	−42	52	7.2	−34	−42	56	6.3	−36	−42	52
	L					6.8	−61	−31	31	3.8	−50	−36	22
	R					4.6	32	−37	39				
	R					6.8	57	−29	33				
Precuneus	L					3.4	−24	−62	34				
Fusiform gyrus	L					4.7	−46	−49	−14	4.7	−46	−49	−14
	R	3.8	46	−47	−14	5.6	46	−45	−13	3.8	46	−47	−14
Middle occipital gyrus	L					7.8	−46	−72	5				
Superior temporal gyrus	L									4.2	−51	10	1
	R	4.7	36	5	−17	6.2	36	−3	−17	4.6	36	3	−17
Middle temporal gyrus	L	9.8	−57	−62	1	7.5	−53	−66	11	7.0	−55	−62	7
	R					8.9	50	−60	3				
	R					4.7	46	−28	−7				
Inferior temporal gyrus	L	5.1	−44	−45	−15					7.3	−48	−70	2
	R	7.6	48	−62	−2	8.4	46	−72	0	7.5	48	−62	−2
Parahippocampal gyrus/amygdala	R					3.5	28	−7	−16				
Uncus/amygdala	L					4.5	−26	−5	−20				
Uncus	L					4.1	−26	−6	−33				
Between caudate/caudate tail and PHG	L	4.3	−36	−14	−9					4.2	−36	−14	−9
Brainstem/midbrain	L	4.4	−2	−27	1	4.8	−6	−6	−8				
	L					4.2	−8	−18	−8				
	R	3.9	6	−18	−1								
Brainstem/pons	R					4.0	8	−33	−29				
Lentiform nucleus/medial globus pallidus	L	4.3	−12	2	2								
Lentiform nucleus/lateral globus pallidus	R	5.6	12	6	−2								
Thalamus	L	3.8	−12	−15	12								
Thalamus/pulvinar	L					4.0	−14	−25	3				
Between thalamus and caudate head	R					3.9	8	0	4	3.9	8	0	4
Cerebellum/anterior lobe/culmen	L	4.3	−38	−52	−28	4.8	−30	−48	−28				
	R	4.3	40	−48	−26	4.7	32	−48	−26	4.2	38	−48	−26
Cerebellum/posterior lobe/declive	L	4.5	−14	−71	−17	6.5	−20	−65	−20	4.4	−14	−71	−17
	L	4.4	−22	−67	−22	4.0	−34	−59	−22	4.4	−22	−67	−22
	R	3.8	38	−57	−21					3.8	38	−57	−21
Cerebellum/posterior lobe/tuber	R	3.5	44	−56	−26								
Cerebellum/posterior lobe/uvula	L									3.4	−32	−61	−24

*Note*: Anatomical regions, peak activation *t*‐values and Talairach‐coordinates for contrast reactive aggressive (a) versus social positive (p) scenarios, separately for violent individuals (VIOL) and control participants (CON) and conjunction{null} analyses including contrast a > p of both groups; H = hemisphere: L = left, R = right, all statistics *p* < .001, uncorrected, minimum voxel cluster size *k *= 10 voxels.

#### 3.3.2 Social positive (P) versus reactive aggressive (A) interaction scenarios

At iso‐cortical level, in both groups the contrast P versus A revealed distributed activation patterns in premotor, medial parietal (particularly in precuneus) and occipital (particularly in cuneus), occipito‐temporal (left fusiform gyrus) and temporal brain regions (for illustration, see Figure 3b, left and right panels; for an overview of recruited brain regions, see Table 2, lower part; and for a detailed list of activation foci, coordinates and *t*‐values, see Table 4). At the sub‐cortical level, both groups showed activation patterns in the cingulate cortex (particularly right lateral in the middle part and bilateral in the posterior part) and in the parahippocampal gyrus. VIOL individuals also recruited areas in the postcentral gyrus, the insula and the anterior cingulate.

**TABLE 4:** Detailed fMRI data for contrast P versus A

BOLD contrast: Social positive versus reactive aggressive condition (p > a)													
	VIOL					CON				VIOL and CON			
Anatomical region	H	t	x	y	z	t	x	y	z	t	x	y	z
Precentral gyrus	L	4.4	−40	−12	63								
	L	4.3	−34	−19	56								
	R	4.3	57	−7	10								
	R	4.3	38	−17	58								
	R	4.0	28	−23	51								
Superior frontal gyrus	L					3.6	−22	39	48				
	R	5.5	30	32	48	4.5	30	26	50	4.4	30	28	50
	R					4.2	36	22	54				
Medial frontal gyrus	L					4.1	−12	36	−12				
	R	3.8	8	54	−3	4.4	6	34	−12				
	R					3.9	6	54	−4				
Middle frontal gyrus	L	3.7	−38	19	27	4.4	−24	28	−15				
	L					4.2	−36	14	55				
	R	6.1	26	27	41	3.7	30	12	53	3.7	28	14	47
	R	4.0	30	10	47	3.6	26	23	41	3.6	26	23	41
	R					3.9	50	36	29				
	R					3.6	50	42	20				
Anterior cingulate	R	3.5	12	48	−6								
Cingulate gyrus	R	3.6	14	−43	33	5.9	6	−39	41	5.7	2	−41	41
Posterior cingulate	L	8.6	−18	−56	14	8.6	−18	−56	12	8.4	−18	−56	14
	R	7.3	16	−51	21	8.1	−14	−52	6	7.0	12	−48	10
	R					7.8	8	−48	8				
Insula	L	5.0	−40	−13	17								
	R	4.2	42	−11	19								
	R	4.1	34	−21	12								
Postcentral gyrus	R	4.0	53	−7	17								
Inferior parietal lobule	R	5.6	42	−68	38					5.6	42	−68	38
Precuneus	L	7.3	−16	−61	25	7.2	−34	−78	39	4.6	−6	−50	47
	L	7.0	−2	−43	43	4.6	−6	−50	47	5.6	−42	−72	44
	L	6.4	−6	−50	47								
	L	5.6	−42	−72	44								
	R					7.1	40	−72	39				
Superior occipital gyrus	L	3.9	−36	−80	28					3.9	−36	−80	28
Cuneus	L	7.8	−10	−97	3	8.0	−6	−99	7	7.7	−8	−99	5
	L					5.5	−30	−86	34				
	R									7.2	10	−92	23
	R									6.9	12	−87	6
Lingual gyrus	L	7.5	−14	−91	−2								
	R					8.9	12	−89	4				
Fusiform gyrus	L	7.9	−30	−37	−12	8.1	−30	−37	−12	7.9	−30	−37	−12
Superior temporal gyrus	L					5.7	−42	−29	7	3.6	−57	−10	−1
	R					4.1	46	−21	8	4.1	46	−21	8
	R	4.5	44	−19	8	3.8	63	−14	1	3.8	63	−14	1
Middle temporal gyrus	L	3.8	−55	−12	−3	4.0	−57	−8	−3				
	R	5.8	59	−4	−5								
Inferior temporal gyrus	L	5.4	−59	−7	−16	4.7	−57	−9	−15	4.7	−57	−9	−15
	R					5.1	53	−7	−20				
Parahippocampal gyrus	R	11.1	28	−41	−6	9.8	28	−41	−8	9.8	28	−41	−8
	R	8.9	30	−28	−19	8.7	30	−28	−20	8.7	30	−28	−20

*Note*: Anatomical regions, peak activation *t*‐values and Talairach‐coordinates for contrast social positive (p) versus reactive aggressive (a) scenarios, separately for violent individuals (VIOL) and control participants (CON), and conjunction{null} analyses including contrast p > a of both groups; H = hemisphere: L = left, R = right, all statistics *p* < .001, uncorrected, minimum voxel cluster size *k* = 10 voxels.

#### 3.3.3 Supplementary analyses

Tables S3.1–S3.4 in Appendix 3 in the Supporting Information show detailed analyses with and without CFT (i.e., intelligence measures) as a covariate. The results were comparable and did not affect the key conclusions of the present study.

Despite the fact that correlation analyses of fMRI data with external variables can at best be seen as an exploratory approach and need to be interpreted with caution due to the correlative nature of the fMRI data, we also provide data on correlation analyses between contrast images (a > p) in emotionally relevant ROIs and both RPQ (total) and valence rating scores (see Appendices 1 and 2 in the Supporting Information). A further discussion of these data would go beyond the scope of the present study; therefore, these data can be used as basis for meta‐analytic research and as an inspiration for future studies on the topic.

## 4 DISCUSSION

In the present study, two groups of participants, that is, individuals, who had committed violent offenses, and control individuals, who had not, were examined using fMRI while watching stimuli selected from a stimulus video inventory developed by Fehr et al. (2014) including quasi‐realistic scenarios showing neutral, positive and reactive aggressive social interactions from a first‐person perspective (Fehr, 2012; Fehr et al., 2014). The study aimed at exploring the neurobiological functioning of individuals with a history of violent offenses within this experimental setting designed to reach high ecological validity using realistic emotional interaction scenarios and a first‐person perspective. Statistical thresholds were set according to the suggestions by Lieberman and Cunningham (2009) to reduce the risk of beta error bias in the exploration of fMRI‐activation patterns that should be used as a hypothetical basis in consecutive studies.

For the identification of overlapping recruitment of brain areas in both groups, we calculated conjunction analyses for the contrasts of interest, that is, reactive aggressive versus positive and positive versus reactive aggressive scenarios. The results in conjunction analyses were only considered for interpretation when both groups also showed significant regional activation foci in the respective group‐related analyses. Activation clusters were interpreted as potentially group‐specific when there were significant activation foci in group‐related analyses, but not in the respective conjunction analyses including both groups. The present fMRI‐analyses were focused on contrasts between reactive aggressive and social positive scenarios.

### 4.1 Behavioural data and the validity of the experimental approach

While there were no group differences in intensity ratings of the scenarios, valence ratings differed between groups: VIOL rated reactive aggressive scenarios less negative than CON. This finding might indicate a reduced fear response in violent individuals on a behavioural level, which might result from an amygdala hypoactivation in this group (cf. Lozier et al., 2014; Rosell & Siever, 2015). This interpretation is supported by our neurobiological findings revealed by the contrast reactive aggressive versus positive interaction, indicating amygdala involvement in CON but not in VIOL participants (see Section 4.2). Moreover, there were pronounced, yet nonsignificant differences between groups: VIOL rated neutral scenarios more negative and social positive scenarios less positive in comparison to CON. Thus, VIOL may have experienced nonthreatening social interaction scenarios more negative than nonviolent individuals, which might express a general mistrust toward social interactions possibly due to life‐long negative experiences (cf. Afifi et al., 2019). However, VIOL compared to CON produced larger distributions in valence ratings for all scenario‐types also indicating more heterogeneous, and thus idiosyncratic, emotional processing.

Summing up, behavioural data confirmed the validity of the applied stimuli. In accordance with the taxonomy of Bradley and Lang (1994), the evaluation of the stimuli revealed higher intensity ratings for social positive and reactive aggressive as compared to neutral scenarios. Reactive aggressive scenarios were rated most intense. Valence ratings revealed positive ratings for positive, negative ratings for reactive aggressive and ratings in‐between for neutral scenarios in both groups.

### 4.2 Neural correlates of reactive aggressive versus social positive scenarios

At a cortical level, we found a large overlap between VIOL and CON in precentral gyrus, premotor, perceptual parieto‐temporal and occipito‐temporal brain regions. Taken together with the findings by Fehr et al. (2014), the stimulus material seems to validly provoke activation in previously proposed experience‐related, context dependent action‐cycle systems. Additionally, both groups showed insula and postcentral gyrus involvement, which have previously been associated with mental states of aversion and pain expectancy (Decety, 2010; Fan et al., 2011; Gu et al., 2010). Contrary to our expectations, we did not find a recruitment of right inferior‐frontal regions previously discussed as an inhibiting instance (cf. Fehr et al., 2014). Neither did we find support for differential patterns of activation in experience‐related action‐perception cycles (Fehr et al., 2014) in violent individuals. Thus, our findings do not support the idea of neuro‐moral or top‐down regulatory cortical dysfunction as has been associated with the proposed proactive and reactive aggressive phenotypes, respectively. This might add to the idea that this distinction might be limited in its explanatory power of violence. However, using this paradigm without a behavioural regulation or decision‐making component, we might not have targeted potentially existent cortical alterations in functioning. Also, cortical structures, which have been implicated in the regulation of negative emotions, have also been strongly associated with the processing of positive emotions or reward as, for example, the orbitofrontal cortex (Hiser & Koenigs, 2018). Thus, null findings might also be the result of comparing the possibly equally strong recruitment of cortical structures during the aggressive and positive scenarios in this study.

The following activation patterns were found in the midbrain and brainstem: CON showed rostral midbrain (PAG) activation and activations in the reticular formation (see Siegel & Victoroff, 2009), whereas VIOL showed distributed caudal midbrain (PAG) involvement. The PAG of the midbrain is assumed to be a key structure in the neural processing and initiation of defence‐, flight‐ and fight‐related behaviours (Roelofs, 2017). Caudal PAG involvement in VIOL individuals fits in well with the proposed function of this subdivision initiating fight and flight reactions by studies investigating both, humans (Roelofs, 2017) and animals (Depaulis et al., 1992). In contrast, CON showed rostral midbrain activation associated with PAG, which was implicated in the initiation of freezing (Roelofs, 2017). Thus, these results support the notion that differences in PAG recruitment might contribute to violent individuals rather “taking action” instead of freezing in threatening situations, and thus may play an important role in the execution of violent behaviour.

Furthermore, the present data indicate activation patterns related to the amygdaloid complex only in the CON but not in the VIOL group, that is, suggesting hypoactivation rather than hyperactivation of the amygdala in this sample of violent offenders, as had been linked to the proactive aggressive phenotype. This is in line with the findings in similar samples, for example, in chronic offenders (e.g., Raine, 2019), and underlines the possible relevance of amygdala hyporeactivity in the occurrence of severe forms of violence. Importantly, this might be the case even in situations where reactive aggression is provoked. Reactive aggression has previously rather been associated with amygdala hyperreactivity, for example, in a study applying a money‐subtraction paradigm in violent offenders (da Cunha‐Bang et al., 2017). The diverging findings in violent offenders might be explained by the use of different experimental approaches, which possibly investigate different kinds of reactive aggression (tit for tat versus defence‐like aggression in a situation more closely resembling threat). However, the findings still remain somewhat unclear and support the idea of going beyond an either/or distinction between reactive and proactive aggression in order to investigate the occurrence of violence. Moreover, as the activation of the amygdaloidal complex during threatening situations was found to be a prerequisite for projecting to the rostral PAG to initiate freezing (Roelofs, 2017), reduced amygdala activation in VIOL might explain the lack of rostral PAG activation and consequently, a reduced ability to freeze in threatening situations. However, it is also possible that VIOL individuals’ amygdala activation was comparably high during both positive and reactive aggressive interaction scenarios and would therefore not reveal neurofunctional differences in the respective contrast. Yet, VIOL individuals’ valence ratings indicated that they perceived aggressive scenarios as negative and positive videos as positive (although the difference between positive and aggressive scenarios was smaller in VIOL than in CON). Moreover, in combination with the revealed PAG activation during the aggressive versus positive contrast, the interpretation of an amygdala hypoactivation during threatening situations possibly contributing to a tendency toward fight and flight reactions seems plausible.

In addition, VIOL seemed to link midbrain behavioural impulses to basal ganglia structures such as the globus pallidus, and therefore might run the risk to perform sub‐cortically driven behaviours that are not justified by emotional and/or contextual adequacy via limbic structures such as the amygdala. In sum, our findings concerning the reactive aggressive versus positive contrast give rise to the assumption of a differential activation pattern in individuals with a history of violent offenses in structures involved in the evaluation of emotional stimuli (amygdala) and in the preparation of defence and action (PAG, basal ganglia), which might enhance fast and potentially impulsive behavioural reactions. This might also indicate a reduced fear response in individuals exhibiting violence.

### 4.3 Neural correlates of social positive scenarios versus reactive aggressive

Contrasting social positive and reactive aggressive scenarios revealed highly reliable activation patterns predominantly distributed in medial parieto‐occipital (precuneus and cuneus) and parieto‐temporal brain regions, parahippocampal areas and premotor cortex. This pattern of activation might reflect a perception‐action network related to episodic memories on stereotypic positive scenarios associated with respective pro‐social behaviours as already shown by Fehr et al. (2014).

Interestingly, VIOL likewise showed activation patterns in the insula (at other sites compared to those activated by reactive aggressive scenarios in both groups), anterior cingulate and postcentral areas. In concert with less positive and more heterogeneous valence‐ratings for social positive scenarios in the VIOL group, this might indicate a somewhat ambivalent psychological and neural processing style of actually positive social interactions in individuals with a history of violent offenses. This might be caused by negative experiences with positive social events during life‐long individual socialization potentially forming traits of conflict mal‐evaluation and respective inadequate violent behaviours (cmp. Fehr, 2012; Lawrence & Hodgkins, 2009). Moreover, VIOL individuals might have experienced positive social interactions as more salient or unexpected compared to aggressive ones. Insula and anterior cingulate cortex activation have been associated with processing salience and prediction errors (Uddin, 2015; Jahn et al., 2014), that is, processing stimuli which stand out or deviate from one's expectations, respectively. To violent individuals, positive social interactions might stand out in which these types of interactions might contradict life‐long experiences and learned expectations (see Afifi et al., 2019). This could represent a neural correlate of the hostile attribution bias, which has been related to reactive aggression (Card & Little, 2006).

### 4.4 Final conclusion

First, the present data regarding the contrast between reactive aggressive and social positive interaction scenarios indicated an involvement of the amygdala in control participants, but not in violent individuals in the context of interpersonal provocation. However, as our findings also hint at a somewhat ambivalent processing style of positive interaction scenarios in violent offenders, when considering our within‐group comparisons, we cannot preclude the possibility that amygdala activation is high in violent individuals during both scenarios. Yet, the specific PAG recruitment in violent offenders revealed by the reactive aggressive versus positive contrast renders this interpretation rather unlikely. Thus, our findings point to a hypoactivation of the amygdala (see Raine, 2019) playing a significant role in violence even when the situation is provocative, that is, usually eliciting reactive aggression, which has previously been linked with amygdala hyperactivation. In line with Raine, our findings point to the need to see beyond the theoretical distinction between proactive and reactive aggression in order to take a step toward understanding the complexity of violence in the real life.

Second, in VIOL participants the caudal part, while in CON participants the rostral part of the midbrain, associated with the PAG, was recruited during the processing of reactive aggressive scenarios. Based on the model described by Roelofs (2017), we concluded that in violent individuals midbrain areas related to fight or flight reactions are involved, whereas in nonviolent individuals midbrain areas related to the inhibition of fight and flight reactions and the initiation of freezing are recruited during the processing of proximally threatening contexts.

Third, based on the contrast between social positive and reactive aggressive interaction scenarios, insula recruitment in the VIOL group together with larger distribution and lower positivity ratings for social positive interaction stimuli led us to conclude that positive interactions might be experienced somewhat ambivalently by violent individuals, which might be caused by a general distrust in social interactions (cf. Lawrence & Hodgkins, 2009) and result in the tendency to interpret social interactions more negatively and provokingly. Thus, these findings argue in favor of a hostile attribution bias in violent individuals (cf. Card & Little, 2006), which might not necessarily be characterized by amygdala hyperactivation as has been proposed to underlie aggression in the reactive aggressive phenotype (Rosell & Siever, 2015). However, insula and anterior cortex recruitment indicated by this contrast might also point to violent individuals experiencing positive social interactions as more salient in comparison with negative interaction scenarios (cf., Uddin, 2015). This might be due to expectancies or schemata that social interactions turn out negatively and that positive social interactions are unlikely to happen due to repeated past and current negative interpersonal experiences in these individuals (see Taubner et al., 2017).

### 4.5 Limitations and perspectives

Contrary to the idea of a neuro‐moral or top‐down regulatory dysfunction in violent individuals (cf. Raine, 2019), we did not find differential activation patterns in VIOL and CON at a cortical level. As this might be related to our experimental design, the validity of the present experimental approach might benefit from adding a decisional component.

The sample size of N = 25 violent and N = 21 nonviolent individuals in the study was low, which limits the explanatory power as well as the generalizability of findings.

Future studies should aim for experimental designs that enable the investigation of different conflict‐related interactions such as relational, pro‐active, instrumental and security‐related aggression (cf. Blair, 2010) in order to gain further insight in the usefulness of a distinction between aggression types on the level of the individual or rather identify other relevant factors (e.g., context‐dependency, rather than dependency on individual characteristics; cf. Fehr & Achtziger, 2021).

Therapeutic interventions might benefit from training violent individuals in being able to establish a “freezing phase” during situations, which are experienced as threatening, as is promoted in mentalization‐based therapy (Bateman & Fonagy, 2013).

## CONFLICT OF INTEREST

The authors declare no conflict of interest.

## AUTHOR CONTRIBUTIONS

*Conception and design*: Svenja Taubner, Thorsten Fehr and Gerhard Roth. *Execution*: Svenja Taubner, David Wisniewski and Silke Wolter. *Analysis*: Thorsten Fehr. *Interpretation of data and writing of original draft*: Sophie Hauschild, Svenja Taubner and Thorsten Fehr. *Review & Editing*: David Wisniewski and Gerhard Roth. *Funding Acquisition*: Svenja Taubner and Gerhard Roth.

### PEER REVIEW

The peer review history for this article is available at https://publons.com/publon/10.1002/brb3.2400

## Supporting information



Supporting information

## Data Availability

Data available offline at the last authors’ research institute due to privacy/ethical restrictions.
