# Supplementary material for: Neural response to aggressive and positive interactions in violent offenders and nonviolent individuals
Source: Brain Behav. 2021 Nov 10;11(12):e32400. doi: 10.1002/brb3.2400 (PMC8671790; doi:10.1002/brb3.2400)
Supplement: Supplementary file 1 — Supporting information [file BRB3-11-e32400-s001.doc]

# Supplementary online material

The following appendices shall on the one hand inspire future research on the topic by additional correlation analyses between contrast images and the covariates RPQt (see appendix 1) and average individual valence ratings on reactive aggressive video scenarios (see appendix 2) and on the other hand the original contrasts a>p and p>a were modelled without and with a covariate CFT (i.e. IQ measures; appendix 3) to disclose a bias derived from intelligence level differences between groups on the discussion of the data in the main report. Correlation analyses based on fMRI-contrast data have to be interpreted with caution as the fMRI data represent differences between beta weights resulting from GLM analyses on modelled data, and thus representing correlative information and not signal intensity data such as for example received from EEG-ERP approaches. Parameter estimates, which are sometimes reported as correlation parameters, also do not reflect signal intensity, but a correlative relationship between data and model predictor (based on the convolution with a reference function, such as the standard hemodynamic response function) respectively defined in a design matrix. Percent signal change (PSC) values were also extraced in several studies and reported as signal intensity equivalent. However, PSC-values are heavily contaminated by the temporal convolution of signals, in particular in event-related designs, because of the inert nature of the underlying BOLD-signal. Hence, as the strength of fMRI analyses is contrasting, we decided to perform exploratory correlation analyses between external parameters (i.e., RPQt and average valence ratings) and regions of interest (ROI) related fMRI-data and contrast images. Emotionally relevant ROIs were selected based on discussions in the corresponding literature (i.e., areas in the limbic system, midbrain, and brainstem).

Appendix 1 – Correlations between contrast images (a>p) and RPQ (total) scores

The following tables contain exploratory data based on correlations between contrast images reactive aggressive vs. social positive (a>p) video scenarios and RPQ total values in selected ROIs.

| **Correlation: (reactive agressive vs. social positive) X RPQ (total)** | | | | | | | | | | | | | | | | | | | | | | | | | | |
| --- | --- | --- | --- | --- | --- | --- | --- | --- | --- | --- | --- | --- | --- | --- | --- | --- | --- | --- | --- | --- | --- | --- | --- | --- | --- | --- |
|  |  | | **VIOL** | | | | | | | | **CON** | | | | | | | | **VIOL + CON** | | | | | | | |
| **anatomical region (ROI)** | **H** | | **t** | | **x** | | **y** | | **z** | | **t** | | **x** | | **y** | | **z** | | **t** | | **x** | | **y** | | **z** | |
|  |  | |  | |  | |  | |  | |  | |  | |  | |  | |  | |  | |  | |  | |
|  | | **positive correlation** | | | | | | | | | | | | | | | | | | | | | | | | |
|  |  | |  | |  | |  | |  | |  | |  | |  | |  | |  | |  | |  | |  | |
| **Orbital Gyrus** | | **L** | |  | |  | |  | |  | | 2.9 | | -30 | | 51 | | -34 | |  | |  | |  | |  |
| **Insula** | | **L** | | 2.5 | | -38 | | 16 | | -1 | | 3.0 | | -38 | | -18 | | -4 | |  | |  | |  | |  |
|  | | **L** | |  | |  | |  | |  | | 2.4 | | -46 | | -22 | | 18 | |  | |  | |  | |  |
| **Anterior Cingulate** | |  | |  | |  | |  | |  | |  | |  | |  | |  | |  | |  | |  | |  |
| **Amygdala** | | **L** | |  | |  | |  | |  | | 2.0 | | -24 | | -5 | | -17 | |  | |  | |  | |  |
| **Mammillary Body** | | **R** | | 1.9 | | 4 | | -10 | | -5 | |  | |  | |  | |  | |  | |  | |  | |  |
| **Thalamus** | | **L** | | 4.2 | | -4 | | -4 | | 6 | |  | |  | |  | |  | |  | |  | |  | |  |
|  | | **L** | | 3.7 | | -12 | | -13 | | 4 | |  | |  | |  | |  | |  | |  | |  | |  |
|  | | **R** | | 3.8 | | 4 | | -9 | | 12 | |  | |  | |  | |  | | 3.0 | | 4 | | -9 | | 12 |
|  | | **R** | | 3.7 | | 16 | | -9 | | 13 | |  | |  | |  | |  | |  | |  | |  | |  |
| **between Thalamus and Midbrain** | | **R** | | 2.1 | | 6 | | -23 | | 1 | |  | |  | |  | |  | |  | |  | |  | |  |
| **Midbrain** | | **R** | |  | |  | |  | |  | |  | |  | |  | |  | | 3.0 | | 2 | | -23 | | 3 |
| **Pons** | | **R** | | 2.7 | | 16 | | -24 | | -22 | | 2.3 | | 10 | | -21 | | -36 | |  | |  | |  | |  |
|  |  | |  | |  | |  | |  | |  | |  | |  | |  | |  | |  | |  | |  | |
|  | | **negative correlation** | | | | | | | | | | | | | | | | | | | | | | | | |
|  |  | |  | |  | |  | |  | |  | |  | |  | |  | |  | |  | |  | |  | |
| **Orbital Gyrus** | | **L** | | 2.3 | | -2 | | 44 | | -19 | |  | |  | |  | |  | |  | |  | |  | |  |
| **Insula** | | **L** | |  | |  | |  | |  | | 4.5 | | -38 | | 20 | | 16 | |  | |  | |  | |  |
|  | | **L** | |  | |  | |  | |  | | 1.9 | | -36 | | 15 | | -6 | |  | |  | |  | |  |
| **Anterior Cingulate** | | **L** | | 2.1 | | -24 | | 39 | | 4 | |  | |  | |  | |  | |  | |  | |  | |  |
|  | | **L** | | 2.1 | | -12 | | 31 | | -8 | |  | |  | |  | |  | |  | |  | |  | |  |
|  | | **L** | | 2.1 | | -2 | | 19 | | -4 | |  | |  | |  | |  | |  | |  | |  | |  |
|  | | **R** | |  | |  | |  | |  | | 5.1 | | 6 | | 21 | | -4 | |  | |  | |  | |  |
| **Hypothalamus** | | **L** | |  | |  | |  | |  | |  | |  | |  | |  | | 2.3 | | -6 | | -4 | | -8 |
| **Midbrain** | | **B** | |  | |  | |  | |  | | 2.4 | | 0 | | -28 | | -14 | |  | |  | |  | |  |
|  | | **L** | |  | |  | |  | |  | | 2.3 | | -6 | | -13 | | -18 | |  | |  | |  | |  |
|  | | **R** | |  | |  | |  | |  | | 2.5 | | 12 | | -16 | | -18 | |  | |  | |  | |  |
|  | | **R** | |  | |  | |  | |  | | 2.1 | | 2 | | -12 | | -16 | |  | |  | |  | |  |
| **Pons** | | **L** | |  | |  | |  | |  | | 2.3 | | -20 | | -29 | | -27 | | 2.2 | | -8 | | -17 | | -21 |
|  | | **L** | |  | |  | |  | |  | | 1.9 | | -8 | | -30 | | -25 | |  | |  | |  | |  |
|  | | **R** | |  | |  | |  | |  | |  | |  | |  | |  | | 2.1 | | 20 | | -42 | | -26 |

**Table S1:** Anatomical regions (Regions Of Interest = ROIs), peak activation t-values, and Talairach-coordinates for correlation analyses between the contrast reactive aggressive vs. social positive scenarios (a>p) and RPQ total scores (RPQt), separately for VIOL (n=25 participants, CON (n = 21 participants), and all (n = 46, all participants pooled) participants; H = hemisphere: L = left, R = right, all statistics p < .05, uncorrected, minimum voxel cluster size k = 10 voxels.

**Appendix 2 – Correlations between contrast images (a>p) and valence ratings**

The following tables contain exploratory data based on correlations between contrast images reactive aggressive vs. social positive (a>p) video scenarios and two kinds of valence parameters. First analyses included average valence ratings on reactive aggressive scenarios (Table S2.1) and second analyses included difference values between average valence ratings on reactive aggressive and social positive scenarios (Table S2.2). One has to consider that valence ratings are always modulated by cognitive top-down processing, whereas just watching scenarios can trigger both automatic bottom-up and top-down processing (depending on individual learning history and emotio-cognitive mental strategies, i.e., Fehr, 2012; Fehr, 2013; Fehr & Herrmann, 2015). This can suppress brain activations that would be triggered during spontaneous and unmodulated processing of the same stimuli. Thus, a different kind of cerebral processing of the same stimuli might have biased the ‘direct’ relationship of different measures while correlated via statistical procedures. Furthermore, VIOL participants might have modulated their responses as they potentially wanted to leave back a good impression, which might have lowered, or at least diffused, the group-specific compliance and valence profile to a certain amount. Future studies might consider nonparametric procedures, nonlinear, or at least, procedures including curve linear model functions. The present state in science on the topic is still on an exploratory level, and we hope to make a substantial contribution to it.

| **Correlation: (reactive agressive vs. social positive) X average valence ratings (a)** | | | | | | | | | | | | | | | | | | | | | | | | | | |
| --- | --- | --- | --- | --- | --- | --- | --- | --- | --- | --- | --- | --- | --- | --- | --- | --- | --- | --- | --- | --- | --- | --- | --- | --- | --- | --- |
|  |  | | **VIOL*** | | | | | | | | **CON*** | | | | | | | | **VIOL* + CON*** | | | | | | | |
| **anatomical region (ROI)** | **H** | | **t** | | **x** | | **y** | | **z** | | **t** | | **x** | | **y** | | **z** | | **t** | | **x** | | **y** | | **z** | |
|  |  | |  | |  | |  | |  | |  | |  | |  | |  | |  | |  | |  | |  | |
|  | | **positive correlation** | | | | | | | | | | | | | | | | | | | | | | | | |
|  |  | |  | |  | |  | |  | |  | |  | |  | |  | |  | |  | |  | |  | |
| **between Claustrum and Insula** | | **L** | | 2.0 | | -32 | | -1 | | 9 | |  | |  | |  | |  | |  | |  | |  | |  |
| **Insula** | | **L** | | 3.8 | | -30 | | 20 | | 8 | |  | |  | |  | |  | | 1.8 | | -30 | | -17 | | 17 |
|  | | **L** | | 3.0 | | -38 | | 8 | | -2 | |  | |  | |  | |  | | 2.8 | | -36 | | 10 | | -2 |
|  | | **L** | | 2.6 | | -30 | | 23 | | -1 | |  | |  | |  | |  | | 2.8 | | -30 | | 20 | | 8 |
|  | | **R** | | 3.3 | | 32 | | 18 | | 5 | |  | |  | |  | |  | | 3.0 | | 38 | | -27 | | 3 |
|  | | **R** | | 2.7 | | 44 | | -16 | | 1 | |  | |  | |  | |  | | 2.2 | | 42 | | -13 | | 15 |
|  | | **R** | |  | |  | |  | |  | |  | |  | |  | |  | | 3.0 | | 38 | | 15 | | -2 |
|  | | **R** | |  | |  | |  | |  | |  | |  | |  | |  | | 1.7 | | 40 | | 14 | | 9 |
|  | | **R** | |  | |  | |  | |  | |  | |  | |  | |  | | 2.1 | | 44 | | -14 | | 1 |
|  | | **R** | |  | |  | |  | |  | |  | |  | |  | |  | | 1.9 | | 26 | | -40 | | 19 |
| **Anterior Cingulate** | | **L** | | 2.3 | | -10 | | 36 | | 20 | | 2.8 | | -8 | | 43 | | 13 | | 2.7 | | -10 | | 34 | | 20 |
|  | | **L** | |  | |  | |  | |  | | 2.3 | | -8 | | 30 | | 22 | | 2.6 | | -12 | | 37 | | -4 |
|  | | **L** | |  | |  | |  | |  | | 2.9 | | -14 | | 39 | | -5 | |  | |  | |  | |  |
|  | | **R** | | 2.0 | | 0 | | 16 | | 14 | | 2.6 | | 18 | | 45 | | 7 | | 3.0 | | 12 | | 35 | | -5 |
|  | | **R** | |  | |  | |  | |  | | 3.3 | | 6 | | 20 | | 17 | | 2.6 | | 2 | | 41 | | 5 |
|  | | **R** | |  | |  | |  | |  | |  | |  | |  | |  | | 2.1 | | 2 | | 20 | | 14 |
| **Amygdala** | | **R** | | 2.1 | | 24 | | -10 | | -8 | |  | |  | |  | |  | | 2.3 | | 22 | | -6 | | -11 |
| **Thalamus** | | **L** | | 2.2 | | -2 | | -6 | | 6 | |  | |  | |  | |  | | 2.1 | | -2 | | -6 | | 6 |
|  | | **R** | | 2.3 | | 2 | | -20 | | 18 | |  | |  | |  | |  | | 3.1 | | 24 | | -19 | | 18 |
|  | | **R** | | 2.6 | | 24 | | -19 | | 18 | |  | |  | |  | |  | |  | |  | |  | |  |
|  | | **R** | | 2.0 | | 20 | | -17 | | 10 | |  | |  | |  | |  | |  | |  | |  | |  |
| **between Anterior Cingulate and Hypothalamus** | | **R** | |  | |  | |  | |  | | 2.1 | | 4 | | 0 | | -8 | |  | |  | |  | |  |
| **Midbrain** | | **L** | |  | |  | |  | |  | |  | |  | |  | |  | | 2.6 | | -18 | | -22 | | -4 |
|  | | **R** | |  | |  | |  | |  | | 2.1 | | 8 | | -18 | | -14 | |  | |  | |  | |  |
| **Pons** | | **L** | |  | |  | |  | |  | | 5.1 | | -10 | | -35 | | -30 | |  | |  | |  | |  |
|  |  | |  | |  | |  | |  | |  | |  | |  | |  | |  | |  | |  | |  | |
|  | | **negative correlation** | | | | | | | | | | | | | | | | | | | | | | | | |
|  |  | |  | |  | |  | |  | |  | |  | |  | |  | |  | |  | |  | |  | |
| **Orbital Gyrus** | | **L** | | 3.8 | | -4 | | 46 | | -19 | | 2.6 | | -28 | | 47 | | -39 | | 2.9 | | -4 | | 38 | | -20 |
| **between Medial Frontal Gyrus and Orbital G.** | | **L** | |  | |  | |  | |  | |  | |  | |  | |  | | 2.7 | | 0 | | 52 | | -18 |
| **Anterior Cingulate** | | **L** | | 2.5 | | -2 | | 2 | | -3 | |  | |  | |  | |  | |  | |  | |  | |  |
| **Thalamus** | | **L** | | 3.3 | | -10 | | -27 | | 0 | |  | |  | |  | |  | | 2.0 | | -10 | | -27 | | 0 |
| **Midbrain** | | **L** | |  | |  | |  | |  | |  | |  | |  | |  | | 2.4 | | -2 | | -22 | | -4 |
| **Pons** | | **L** | | 2.4 | | -14 | | -21 | | -23 | |  | |  | |  | |  | | 3.3 | | -16 | | -19 | | -23 |
|  | | **L** | | 1.8 | | -8 | | -28 | | -25 | |  | |  | |  | |  | |  | |  | |  | |  |
|  | | **R** | | 2.0 | | 8 | | -29 | | -27 | |  | |  | |  | |  | | 2.5 | | 8 | | -29 | | -27 |
| **Red Nucleus** | | **L** | | 4.2 | | -4 | | -20 | | -2 | |  | |  | |  | |  | |  | |  | |  | |  |

**Table S2.1:** Anatomical regions (Regions Of Interest = ROIs), peak activation t-values, and Talairach-coordinates for correlation analyses between the contrast reactive aggressive vs. social positive scenarios (a>p) and average valence ratings on reactive aggressive scenarios (a), separately for VIOL (n=24* participants, CON (n = 20* participants), and all (n = 46, all participants pooled) participants; H = hemisphere: L = left, R = right, all statistics p < .05, uncorrected, minimum voxel cluster size k = 10 voxels.

| **Correlation: (reactive agressive vs. social positive) X average valence ratings (a-p)** | | | | | | | | | | | | | | | | | | | | | | | | | | |
| --- | --- | --- | --- | --- | --- | --- | --- | --- | --- | --- | --- | --- | --- | --- | --- | --- | --- | --- | --- | --- | --- | --- | --- | --- | --- | --- |
|  |  | | **VIOL** | | | | | | | | **CON** | | | | | | | | **VIOL + CON** | | | | | | | |
| **anatomical region (ROI)** | **H** | | **t** | | **x** | | **y** | | **z** | | **t** | | **x** | | **y** | | **z** | | **t** | | **x** | | **y** | | **z** | |
|  |  | |  | |  | |  | |  | |  | |  | |  | |  | |  | |  | |  | |  | |
|  | | **positive correlation** | | | | | | | | | | | | | | | | | | | | | | | | |
|  |  | |  | |  | |  | |  | |  | |  | |  | |  | |  | |  | |  | |  | |
| **Orbital Gyrus** | | L | |  | |  | |  | |  | | 2.1 | | -28 | | 47 | | -39 | |  | |  | |  | |  |
| **Insula** | | L | |  | |  | |  | |  | |  | |  | |  | |  | | 2.0 | | -42 | | -12 | | -9 |
|  | | R | |  | |  | |  | |  | | 2.1 | | 36 | | 18 | | 8 | | 1.9 | | 46 | | -34 | | 18 |
| **Anterior Cingulate** | | L | | 3.6 | | -2 | | 2 | | -5 | |  | |  | |  | |  | |  | |  | |  | |  |
|  | | L | | 1.8 | | -4 | | 15 | | 23 | |  | |  | |  | |  | |  | |  | |  | |  |
|  | | R | | 1.9 | | 4 | | 15 | | 21 | |  | |  | |  | |  | |  | |  | |  | |  |
| **between Anterior Cingulate and Hypothalamus** | | L | |  | |  | |  | |  | |  | |  | |  | |  | | 2.9 | | -6 | | 0 | | -5 |
| **between Midbrain and Pulvinar** | | L | |  | |  | |  | |  | |  | |  | |  | |  | | 2.2 | | -10 | | -31 | | 0 |
| **Midbrain** | | L | | 3.5 | | -10 | | -29 | | 1 | |  | |  | |  | |  | |  | |  | |  | |  |
| **Pons** | | L | | 2.7 | | -12 | | -17 | | -28 | |  | |  | |  | |  | | 3.7 | | -14 | | -17 | | -26 |
|  | | L | | 2.5 | | -10 | | -30 | | -27 | |  | |  | |  | |  | | 2.1 | | -10 | | -29 | | -27 |
| **Red Nucleus** | | L | | 3.4 | | -6 | | -20 | | -4 | |  | |  | |  | |  | | 2.4 | | -4 | | -22 | | -6 |
|  | | R | | 2.7 | | 8 | | -20 | | -6 | |  | |  | |  | |  | |  | |  | |  | |  |
|  |  | |  | |  | |  | |  | |  | |  | |  | |  | |  | |  | |  | |  | |
|  | | **negative correlation** | | | | | | | | | | | | | | | | | | | | | | | | |
|  |  | |  | |  | |  | |  | |  | |  | |  | |  | |  | |  | |  | |  | |
| **between Insula and Inferior Frontal Gyrus** | | L | | 2.2 | | -38 | | 20 | | 14 | |  | |  | |  | |  | | 2.1 | | -38 | | 20 | | 14 |
|  | | R | | 1.9 | | 34 | | 15 | | -7 | |  | |  | |  | |  | |  | |  | |  | |  |
| **Insula** | | L | | 2.7 | | -36 | | 8 | | 0 | |  | |  | |  | |  | | 2.5 | | -36 | | 8 | | 0 |
|  | | L | | 2.5 | | -30 | | 20 | | 8 | |  | |  | |  | |  | | 2.1 | | -32 | | 22 | | 8 |
|  | | L | |  | |  | |  | |  | |  | |  | |  | |  | | 1.8 | | -42 | | -15 | | 14 |
|  | | R | | 2.4 | | 44 | | -16 | | 1 | | 2.1 | | 32 | | -1 | | 15 | | 3.0 | | 38 | | -25 | | 5 |
|  | | R | | 2.6 | | 32 | | 18 | | 5 | |  | |  | |  | |  | | 2.0 | | 40 | | -15 | | 19 |
| **Anterior Cingulate** | | L | | 2.7 | | -10 | | 37 | | -5 | | 2.7 | | -8 | | 43 | | 13 | | 2.7 | | -8 | | 27 | | -8 |
|  | | L | | 2.6 | | -8 | | 25 | | -6 | | 1.8 | | -6 | | 32 | | 11 | |  | |  | |  | |  |
|  | | R | | 3.4 | | 10 | | 33 | | -7 | | 2.0 | | 6 | | 39 | | 7 | | 4.0 | | 10 | | 35 | | -7 |
|  | | R | | 2.0 | | 2 | | 39 | | 0 | | 2.3 | | 18 | | 45 | | 7 | |  | |  | |  | |  |
|  | | R | |  | |  | |  | |  | | 3.1 | | 6 | | 20 | | 17 | |  | |  | |  | |  |
| **Amygdala** | | L | | 1.9 | | -28 | | -4 | | -12 | |  | |  | |  | |  | |  | |  | |  | |  |
| **Mammillary Body** | | R | | 2.2 | | 4 | | -12 | | -9 | |  | |  | |  | |  | |  | |  | |  | |  |
| **Thalamus** | | R | |  | |  | |  | |  | |  | |  | |  | |  | | 2.3 | | 22 | | -17 | | 6 |
|  | | R | |  | |  | |  | |  | |  | |  | |  | |  | | 2.1 | | 24 | | -19 | | 18 |
| **between Anterior Cingulate and Hypothalamus** | | R | |  | |  | |  | |  | | 2.0 | | 4 | | 0 | | -8 | |  | |  | |  | |  |
| **Midbrain** | | R | |  | |  | |  | |  | |  | |  | |  | |  | | 2.2 | | 4 | | -12 | | -11 |
| **Pons** | | L | |  | |  | |  | |  | | 5.4 | | -10 | | -35 | | -30 | |  | |  | |  | |  |

**Table S2.2:** Anatomical regions (Regions Of Interest = ROIs), peak activation t-values, and Talairach-coordinates for correlation analyses between the contrast reactive aggressive vs. social positive scenarios (a>p) and difference values between average valence ratings on reactive aggressive and social positive scenarios (a-p), separately for VIOL (n=24* participants, CON (n = 20* participants), and all (n = 46, all participants pooled) participants; H = hemisphere: L = left, R = right, all statistics p < .05, uncorrected, minimum voxel cluster size k = 10 voxels.

**Appendix 3 – Contrasting reactive aggressive vs. social positive (a>p) and vice versa (p>a) scenario condition with and without CFT measures as covariate**

| **Contrast: reactive agressive vs. social positive scenario condition** | | | | | | | | | |
| --- | --- | --- | --- | --- | --- | --- | --- | --- | --- |
| **VIOL (n=25)** |  | **a > p** | | | | **a > p (cov CFT)** | | | |
| **anatomical region** | **H** | **t** | **x** | **y** | **z** | **t** | **x** | **y** | **z** |
|  |  |  |  |  |  |  |  |  |  |
| Precentral Gyrus | L | 5.1 | -57 | 12 | 5 | 7.0 | -51 | 6 | 13 |
|  | L |  |  |  |  | 5.8 | -59 | 14 | 7 |
| Superior Frontal Gyrus | L | 6.7 | -16 | 1 | 63 |  |  |  |  |
|  | R | 5.2 | 10 | 9 | 62 | 5.3 | 10 | 9 | 62 |
|  | R | 4.7 | 6 | 18 | 58 | 4.8 | 8 | 17 | 58 |
|  | R | 4.7 | 2 | 3 | 66 |  |  |  |  |
| Middle Frontal Gyrus | L | 4.9 | -24 | -7 | 57 | 7.3 | -22 | -3 | 61 |
|  | L | 4.7 | -40 | 40 | 22 | 4.6 | -40 | 40 | 22 |
|  | L |  |  |  |  | 4.5 | -44 | 38 | 15 |
| Inferior Frontal Gyrus | L | 5.8 | -51 | 8 | 14 | 5.1 | -44 | 17 | -1 |
|  | L | 4.7 | -38 | 15 | -14 | 5.1 | -40 | 15 | -16 |
|  | L | 4.6 | -44 | 17 | -1 | 4.9 | -53 | 9 | 27 |
|  | L | 4.5 | -53 | 9 | 29 | 4.4 | -42 | 35 | 7 |
|  | L | 4.6 | -44 | 35 | 6 |  |  |  |  |
|  | R | 5.5 | 55 | 19 | -1 | 5.8 | 53 | 25 | -6 |
|  | R | 4.7 | 53 | 31 | -7 | 3.7 | 44 | 27 | -1 |
| Cingulate Gyrus | L | 6.5 | -6 | -17 | 40 | 6.9 | -4 | -10 | 37 |
|  | L | 3.8 | -4 | 13 | 27 | 5.1 | -12 | -21 | 38 |
|  | L |  |  |  |  | 4.3 | -6 | 6 | 33 |
|  | L |  |  |  |  | 4.0 | -4 | 11 | 27 |
|  | R | 4.8 | 8 | -17 | 41 | 4.8 | 8 | -17 | 40 |
| Insula | L | 4.6 | -36 | 20 | 3 | 8.0 | -53 | -25 | 40 |
| Postcentral Gyrus | L | 8.0 | -55 | -25 | 40 | 7.2 | -65 | -22 | 21 |
|  | L | 6.8 | -53 | -29 | 51 |  |  |  |  |
|  | R | 6.7 | 63 | -26 | 20 | 4.7 | 30 | -34 | 55 |
|  | R | 4.3 | 34 | -41 | 68 | 3.9 | 34 | -39 | 68 |
|  | R | 3.6 | 34 | -32 | 62 | 7.7 | 63 | -28 | 20 |
| Superior Parietal Lobule | L | 6.8 | -24 | -48 | 59 | 7.3 | -24 | -46 | 58 |
|  | L |  |  |  |  | 6.0 | -36 | -49 | 61 |
|  | R | 5.9 | 32 | -46 | 58 | 8.2 | 30 | -48 | 58 |
| Inferior Parietal Lobule | L | 8.4 | -59 | -33 | 31 | 9.2 | -61 | -31 | 31 |
|  | L | 6.3 | -36 | -42 | 52 | 7.3 | -36 | -38 | 50 |
|  | R |  |  |  |  | 4.7 | 63 | -31 | 31 |
| Precuneus | L |  |  |  |  | 4.8 | -18 | -59 | 55 |
| Fusiform Gyrus | R | 3.8 | 46 | -47 | -14 |  |  |  |  |
| Superior Temporal Gyrus | L |  |  |  |  | 4.8 | -51 | 10 | 1 |
|  | R | 4.7 | 36 | 5 | -17 | 3.9 | 36 | 3 | -20 |
|  | R |  |  |  |  | 3.8 | 38 | 13 | -21 |
| Middle Temporal Gyrus | L | 9.8 | -57 | -62 | 1 | 10.8 | -57 | -62 | 3 |
|  | R |  |  |  |  | 8.5 | 51 | -60 | 5 |
|  | R |  |  |  |  | 4.3 | 50 | -27 | -5 |
| Inferior Temporal Gyrus | L | 5.1 | -44 | -45 | -15 | 6.0 | -44 | -43 | -15 |
|  | R | 7.6 | 48 | -62 | -2 | 7.6 | 51 | -72 | 2 |
|  | R |  |  |  |  | 4.0 | 46 | -43 | -15 |
| between Inferior Temporal Gyrus and Middle Occipital Gyrus | R |  |  |  |  | 8.2 | 46 | -62 | -2 |
| between Caudate/Caudate Tail and Parahippocampal Gyrus | L | 4.3 | -36 | -14 | -9 |  |  |  |  |
| Caudate Tail | L |  |  |  |  | 4.6 | -36 | -16 | -8 |
| Caudate Body | R |  |  |  |  | 6.6 | 12 | 4 | 5 |
| Lateral Globus Pallidus | L |  |  |  |  | 4.8 | -12 | 4 | 3 |
| Lateral Globus Pallidus | R |  |  |  |  | 6.2 | 14 | 6 | -2 |
| Brainstem/Midbrain | L | 4.4 | -2 | -27 | 1 | 4.6 | -10 | -18 | -2 |
|  | L |  |  |  |  | 4.5 | -2 | -27 | 0 |
|  | R | 3.9 | 6 | -18 | -1 |  |  |  |  |
| Lentiform Nucleus/Medial Globus Pallidus | L | 4.3 | -12 | 2 | 2 |  |  |  |  |
|  | R | 5.6 | 12 | 6 | -2 |  |  |  |  |
| Thalamus | L | 3.8 | -12 | -15 | 12 | 5.4 | -12 | -19 | 6 |
|  | R |  |  |  |  | 5.4 | 6 | -17 | 1 |
| Cerebellum/Anterior Lobe/Culmen | L | 4.3 | -38 | -52 | -28 | 5.4 | -36 | -52 | -28 |
|  | L |  |  |  |  | 4.6 | -30 | -58 | -26 |
|  | R | 4.3 | 40 | -48 | -26 | 4.9 | 40 | -46 | -26 |
| Culmen of Vermis | B |  |  |  |  | 4.4 | 0 | -62 | -5 |
| Cerebellum/Posterior Lobe/Declive | L | 4.5 | -14 | -71 | -17 | 5.3 | -22 | -67 | -22 |
|  | L | 4.4 | -22 | -67 | -22 | 5.0 | -14 | -71 | -17 |
|  | R | 3.8 | 38 | -57 | -21 | 4.5 | 26 | -67 | -22 |
|  | R |  |  |  |  | 3.9 | 34 | -57 | -21 |
| Cerebellum/Posterior Lobe/Tuber | R | 3.5 | 44 | -56 | -26 |  |  |  |  |

Table S3.1: Anatomical regions, peak activation t-values, and Talairach-coordinates for contrast reactive aggressive (a) vs. social positive (p) scenarios in the VIOL sub-sample (n = 25), separately without (a > p) and with (a > p (cov CFT)) CFT values as covariate; H = hemisphere: L = left, R = right, all statistics p < .001, uncorrected, minimum voxel cluster size k = 10 voxels.

| **Contrast: reactive agressive vs. social positive scenario condition** | | | | | | | | | |
| --- | --- | --- | --- | --- | --- | --- | --- | --- | --- |
| **CON (n=21)** |  | **a > p** | | | | **a > p (cov CFT)** | | | |
| **anatomical region** | **H** | **t** | **x** | **y** | **z** | **t** | **x** | **y** | **z** |
|  |  |  |  |  |  |  |  |  |  |
| Precentral Gyrus | L |  |  |  |  | 4.8 | -40 | -4 | 44 |
|  | R | 4.0 | 48 | 2 | 37 | 4.5 | 50 | 2 | 37 |
|  | R | 3.5 | 40 | 1 | 26 | 4.0 | 51 | 4 | 44 |
|  |  |  |  |  |  | 5.3 | 2 | 0 | 68 |
| Superior Frontal Gyrus | L | 6.6 | -10 | -3 | 66 | 7.6 | -16 | 3 | 64 |
|  | L | 6.0 | -22 | -5 | 63 | 6.9 | -10 | -4 | 67 |
|  | L | 4.3 | -42 | -4 | 46 |  |  |  |  |
|  | R | 5.1 | 10 | 1 | 68 | 5.2 | 10 | 3 | 70 |
|  | R | 5.0 | 18 | 1 | 64 | 5.2 | 8 | -6 | 70 |
|  | R | 4.3 | 8 | 28 | 50 | 4.7 | 20 | -1 | 65 |
|  | R | 3.4 | 12 | 20 | 51 | 4.4 | 10 | 30 | 50 |
| Medial Frontal Gyrus | L | 3.9 | -8 | 46 | 22 | 4.8 | -10 | 48 | 23 |
| Middle Frontal Gyrus | L |  |  |  |  | 4.8 | -26 | -9 | 59 |
|  | R | 4.3 | 26 | -5 | 59 | 5.8 | 40 | 1 | 50 |
|  | R | 4.7 | 38 | -1 | 50 |  |  |  |  |
| Inferior Frontal Gyrus | L | 6.1 | -53 | 7 | 29 | 5.9 | -53 | 7 | 31 |
|  | L | 5.4 | -53 | 8 | 14 | 4.9 | -46 | 5 | 18 |
|  | L | 4.6 | -30 | 17 | -14 | 4.8 | -30 | 17 | -14 |
|  | L |  |  |  |  | 4.5 | -44 | 31 | 6 |
|  | R | 4.3 | 42 | 25 | 2 | 4.0 | 42 | 0 | 30 |
|  | R | 3.6 | 51 | 17 | -3 | 5.4 | 42 | 29 | 6 |
|  | R |  |  |  |  | 4.8 | 51 | 16 | 1 |
| between Inferior Frontal Gyrus and Precentral G. | L |  |  |  |  | 4.3 | -51 | 12 | 3 |
| between Inferior Frontal Gyrus and Superior Temporal Gyrus | L |  |  |  |  | 4.0 | -30 | 5 | -10 |
| between Insula, Inferior Frontal Gyrus, and Precentral Gyrus | R | 3.5 | 51 | 12 | 3 |  |  |  |  |
| Paracentral Lobule | R |  |  |  |  | 3.7 | 8 | -33 | 70 |
| Cingulate Gyrus | L | 6.8 | -12 | -25 | 40 | 7.4 | -8 | -21 | 40 |
|  | L | 4.7 | -2 | -2 | 33 | 5.9 | -2 | -4 | 35 |
|  | R | 4.6 | 10 | -19 | 40 | 4.5 | 12 | -21 | 42 |
|  | R |  |  |  |  | 4.4 | 8 | -12 | 39 |
| Insula | L | 4.0 | -48 | -36 | 20 |  |  |  |  |
| Postcentral Gyrus | L | 7.3 | -65 | -24 | 21 | 7.3 | -57 | -25 | 42 |
|  | L | 7.0 | -53 | -25 | 40 |  |  |  |  |
|  | L | 6.6 | -61 | -22 | 32 |  |  |  |  |
|  | R | 7.2 | 65 | -28 | 20 | 5.9 | 40 | -39 | 65 |
|  | R |  |  |  |  | 4.5 | 18 | -49 | 69 |
|  | R |  |  |  |  | 4.5 | 8 | -43 | 70 |
| between Postcentral G. and Paracentral Lobule | R | 3.9 | 10 | -39 | 68 |  |  |  |  |
| Superior Parietal Lobule | L | 6.7 | -24 | -51 | 60 | 6.4 | -24 | -48 | 59 |
|  | R | 10.1 | 30 | -46 | 58 |  |  |  |  |
| Inferior Parietal Lobule | L | 7.3 | -57 | -24 | 25 | 7.2 | -57 | -28 | 27 |
|  | L | 7.2 | -34 | -42 | 56 | 6.9 | -38 | -42 | 57 |
|  | L | 6.8 | -61 | -31 | 31 | 6.3 | -38 | -43 | 43 |
|  | R | 4.6 | 32 | -37 | 39 | 8.8 | 32 | -44 | 56 |
|  | R | 6.8 | 57 | -29 | 33 | 8.5 | 59 | -32 | 29 |
|  | R |  |  |  |  | 6.3 | 63 | -28 | 22 |
| Supramarginal Gyrus | L |  |  |  |  | 7.1 | -51 | -37 | 37 |
|  | L |  |  |  |  | 5.7 | -42 | -39 | 35 |
| Precuneus | L | 3.4 | -24 | -62 | 34 | 5.1 | -26 | -44 | 43 |
| Fusiform Gyrus | L | 4.7 | -46 | -49 | -14 | 5.4 | -44 | -53 | -7 |
|  | L |  |  |  |  | 5.0 | -44 | -47 | -16 |
|  | R | 5.6 | 46 | -45 | -13 | 5.6 | 48 | -51 | -9 |
| Middle Occipital Gyrus | L | 7.8 | -46 | -72 | 5 | 7.6 | -46 | -74 | 6 |
|  | L |  |  |  |  | 6.8 | -48 | -72 | -3 |
|  | L |  |  |  |  | 6.2 | -51 | -67 | -10 |
|  | L |  |  |  |  | 5.6 | -42 | -64 | -2 |
| Superior Temporal Gyrus | R | 6.2 | 36 | -3 | -17 | 8.8 | 36 | 1 | -17 |
| Middle Temporal Gyrus | L | 7.5 | -53 | -66 | 11 | 7.0 | -46 | -66 | 11 |
|  | L |  |  |  |  | 6.5 | -55 | -68 | 9 |
|  | R | 8.9 | 50 | -60 | 3 | 9.1 | 50 | -58 | 1 |
|  | R | 4.7 | 46 | -28 | -7 | 8.3 | 44 | -60 | 7 |
|  | R |  |  |  |  | 4.2 | 48 | -44 | 4 |
|  | R |  |  |  |  | 3.8 | 46 | -31 | -5 |
| between Inferior Temporal Gyrus and Middle Temporal Gyrus | R |  |  |  |  | 3.9 | 48 | -30 | -15 |
| Inferior Temporal Gyrus | R | 8.4 | 46 | -72 | 0 | 8.4 | 46 | -72 | 0 |
| Parahippocampal Gyrus/Amygdala | R | 3.5 | 28 | -7 | -16 |  |  |  |  |
| Uncus/Amygdala | L | 4.5 | -26 | -5 | -20 |  |  |  |  |
| Amygdala | L |  |  |  |  | 5.5 | -26 | -7 | -16 |
|  | R |  |  |  |  | 4.9 | 22 | -7 | -18 |
| Uncus | L | 4.1 | -26 | -6 | -33 | 4.3 | -26 | -6 | -33 |
|  | R |  |  |  |  | 4.5 | 30 | -3 | -22 |
| Parahippocampal Gyrus | L |  |  |  |  | 5.4 | -38 | -7 | -15 |
| Brainstem/Midbrain | L | 4.8 | -6 | -6 | -8 | 6.0 | -6 | -8 | -10 |
|  | L | 4.2 | -8 | -18 | -8 | 4.0 | -8 | -22 | -7 |
| Brainstem/Pons | R | 4.0 | 8 | -33 | -29 | 4.0 | 8 | -30 | -27 |
| Thalamus/Pulvinar | L | 4.0 | -14 | -25 | 3 |  |  |  |  |
| Thalamus/Ventral Posterior Medial Nucleus | L |  |  |  |  | 4.1 | -16 | -23 | 3 |
| between Thalamus and Caudate/Caudate Head | R | 3.9 | 8 | 0 | 4 |  |  |  |  |
| Cerebellum/Anterior Lobe/Culmen | L | 4.8 | -30 | -48 | -28 |  |  |  |  |
|  | R | 4.7 | 32 | -48 | -26 | 5.0 | 34 | -48 | -28 |
| Cerebellum/Posterior Lobe/Declive | L | 6.5 | -20 | -65 | -20 | 6.0 | -20 | -65 | -20 |
|  | L | 4.0 | -34 | -59 | -22 |  |  |  |  |
| Cerebellum/Posterior Lobe/Tuber | R |  |  |  |  | 3.9 | 38 | -59 | -24 |
| Precentral Gyrus | L |  |  |  |  | 4.8 | -40 | -4 | 44 |
|  | R | 4.0 | 48 | 2 | 37 | 4.5 | 50 | 2 | 37 |
|  | R | 3.5 | 40 | 1 | 26 | 4.0 | 51 | 4 | 44 |

Table S3.2: Anatomical regions, peak activation t-values, and Talairach-coordinates for contrast reactive aggressive (a) vs. social positive (p) scenarios in the CON sub-sample (n = 21), separately without (a > p) and with (a > p (cov CFT)) CFT values as covariate; H = hemisphere: L = left, R = right, all statistics p < .001, uncorrected, minimum voxel cluster size k = 10 voxels.

| **Contrast: social positive vs. reactive agressive scenario condition** | | | | | | | | | |
| --- | --- | --- | --- | --- | --- | --- | --- | --- | --- |
| **VIOL (n=25)** |  | **p > a** | | | | **p > a (cov CFT)** | | | |
| **anatomical region** | **H** | **t** | **x** | **y** | **z** | **t** | **x** | **y** | **z** |
|  |  |  |  |  |  |  |  |  |  |
| Precentral Gyrus | L | 4.3 | -34 | -19 | 56 | 4.1 | -36 | -19 | 56 |
|  | L | 4.4 | -40 | -12 | 63 | 3.7 | -28 | -20 | 58 |
|  | R | 4.3 | 57 | -7 | 10 | 4.9 | 57 | -7 | 10 |
|  | R | 4.3 | 38 | -17 | 58 | 4.3 | 38 | -14 | 60 |
|  | R | 4.0 | 28 | -23 | 51 |  |  |  |  |
| Superior Frontal Gyrus | R | 5.5 | 30 | 32 | 48 | 5.1 | 28 | 34 | 50 |
|  | R |  |  |  |  | 4.0 | 30 | 12 | 49 |
| Medial Frontal Gyrus | L |  |  |  |  | 5.3 | -14 | 44 | -9 |
|  |  |  |  |  |  | 4.3 | -14 | 36 | -12 |
|  | R | 3.8 | 8 | 54 | -3 |  |  |  |  |
| Middle Frontal Gyrus | L | 3.7 | -38 | 19 | 27 |  |  |  |  |
|  | R | 6.1 | 26 | 27 | 41 | 5.5 | 26 | 27 | 41 |
|  | R | 4.0 | 30 | 10 | 47 | 5.0 | 36 | 30 | 46 |
| Anterior Cingulate | R | 3.5 | 12 | 48 | -6 |  |  |  |  |
| Cingulate Gyrus | R | 3.6 | 14 | -43 | 33 | 5.3 | 8 | -37 | 39 |
| Posterior Cingulate | L | 8.6 | -18 | -56 | 14 | 8.5 | -18 | -56 | 14 |
|  | R | 7.3 | 16 | -51 | 21 |  |  |  |  |
| Insula | L | 5.0 | -40 | -13 | 17 | 4.8 | -40 | -13 | 19 |
|  | R | 4.2 | 42 | -11 | 19 | 4.0 | 44 | -9 | 19 |
|  | R | 4.1 | 34 | -21 | 12 |  |  |  |  |
| Postcentral Gyrus | R | 4.0 | 53 | -7 | 17 | 4.3 | 51 | -9 | 17 |
| Inferior Parietal Lobule | R | 5.6 | 42 | -68 | 38 |  |  |  |  |
| Precuneus | L | 7.3 | -16 | -61 | 25 | 6.4 | -2 | -41 | 43 |
|  | L | 7.0 | -2 | -43 | 43 | 5.9 | -6 | -50 | 47 |
|  | L | 6.4 | -6 | -50 | 47 | 5.4 | -42 | -74 | 42 |
|  | L | 5.6 | -42 | -72 | 44 |  |  |  |  |
|  | R |  |  |  |  | 6.0 | 42 | -70 | 39 |
| Superior Occipital Gyrus | L | 3.9 | -36 | -80 | 28 | 4.1 | -36 | -78 | 30 |
| Cuneus | L | 7.8 | -10 | -97 | 3 | 9.6 | -22 | -97 | 0 |
|  | L |  |  |  |  | 9.5 | -8 | -99 | 5 |
|  | R |  |  |  |  | 11.0 | 12 | -92 | 21 |
|  | R |  |  |  |  | 9.0 | 18 | -95 | 8 |
| Lingual Gyrus | L | 7.5 | -14 | -91 | -2 |  |  |  |  |
| Fusiform Gyrus | L | 7.9 | -30 | -37 | -12 | 4.8 | -59 | -11 | -23 |
| Superior Temporal Gyrus | L |  |  |  |  | 4.1 | -57 | -29 | 7 |
|  | L |  |  |  |  | 4.0 | -57 | -10 | -1 |
|  | R | 4.5 | 44 | -19 | 8 | 5.3 | 61 | -15 | 3 |
|  | R |  |  |  |  | 4.6 | 44 | -19 | 8 |
| Middle Temporal Gyrus | L | 3.8 | -55 | -12 | -3 | 5.0 | -57 | -5 | -15 |
|  | R | 5.8 | 59 | -4 | -5 | 6.8 | 59 | -2 | -7 |
| Inferior Temporal Gyrus | L | 5.4 | -59 | -7 | -16 |  |  |  |  |
| Parahippocampal Gyrus | R | 11.1 | 28 | -41 | -6 | 12.1 | 28 | -39 | -8 |
|  | R | 8.9 | 30 | -28 | -19 | 10.9 | 30 | -28 | -20 |
| between Parahippocampal Gyrus and Culmen | L |  |  |  |  | 8.3 | 16 | -43 | -5 |

Table S3.3: Anatomical regions, peak activation t-values, and Talairach-coordinates for contrast social positive (p) vs. reactive aggressive (a) scenarios in the VIOL sub-sample (n = 25), separately without (p > a) and with (p > a (cov CFT)) CFT values as covariate; H = hemisphere: L = left, R = right, all statistics p < .001, uncorrected, minimum voxel cluster size k = 10 voxels.

| **Contrast: social positive vs. reactive agressive scenario condition** | | | | | | | | | |
| --- | --- | --- | --- | --- | --- | --- | --- | --- | --- |
| **CON (n=21)** |  | **p > a** | | | | **p > a (cov CFT)** | | | |
| **anatomical region** | **H** | **t** | **x** | **y** | **z** | **t** | **x** | **y** | **z** |
|  |  |  |  |  |  |  |  |  |  |
| Superior Frontal Gyrus | L | 3.6 | -22 | 39 | 48 | 5.2 | -12 | 51 | 42 |
|  | R | 4.5 | 30 | 26 | 50 | 7.2 | 24 | 56 | 1 |
|  | R | 4.2 | 36 | 22 | 54 | 4.5 | 24 | 31 | 46 |
| Medial Frontal Gyrus | L | 4.1 | -12 | 36 | -12 |  |  |  |  |
|  | R | 4.4 | 6 | 34 | -12 | 6.1 | 6 | 34 | -13 |
|  | R | 3.9 | 6 | 54 | -4 |  |  |  |  |
| Middle Frontal Gyrus | L | 4.4 | -24 | 28 | -15 | 4.2 | -36 | 14 | 55 |
|  | L | 4.2 | -36 | 14 | 55 |  |  |  |  |
|  | R | 3.7 | 30 | 12 | 53 | 5.7 | 28 | 22 | 56 |
|  | R | 3.6 | 26 | 23 | 41 | 4.8 | 30 | 20 | 47 |
|  | R | 3.9 | 50 | 36 | 29 | 3.7 | 32 | 12 | 55 |
|  | R | 3.6 | 50 | 42 | 20 | 4.5 | 51 | 33 | 30 |
| Inferior Frontal Gyrus | L |  |  |  |  | 4.6 | -28 | 28 | -17 |
| between Inferior Frontal G. and Middle Frontal G. | L |  |  |  |  | 4.5 | -46 | 40 | -15 |
| Cingulate Gyrus | L |  |  |  |  | 5.3 | -4 | -45 | 39 |
|  | R | 5.9 | 6 | -39 | 41 | 6.5 | 2 | -39 | 42 |
| Posterior Cingulate | L | 8.6 | -18 | -56 | 12 | 8.5 | -16 | -56 | 10 |
|  | R | 8.1 | -14 | -52 | 6 | 8.1 | 8 | -57 | 16 |
|  | R | 7.8 | 8 | -48 | 8 | 8.1 | 8 | -48 | 13 |
| Superior Parietal Lobule | L |  |  |  |  | 7.2 | -38 | -70 | 44 |
|  | R |  |  |  |  | 6.5 | 38 | -70 | 46 |
| Angular Gyrus | L |  |  |  |  | 5.4 | -36 | -72 | 31 |
|  | L |  |  |  |  | 4.3 | -50 | -68 | 31 |
|  | R |  |  |  |  | 6.2 | 42 | -61 | 31 |
| Precuneus | L | 7.2 | -34 | -78 | 39 | 7.2 | -34 | -76 | 39 |
|  | L | 4.6 | -6 | -50 | 47 | 5.0 | -6 | -48 | 47 |
|  | R | 7.1 | 40 | -72 | 39 | 4.2 | 8 | -71 | 53 |
| Superior Occipital Gyrus | R |  |  |  |  | 9.2 | 36 | -80 | 33 |
| Cuneus | L | 8.0 | -6 | -99 | 7 | 4.9 | -30 | -86 | 34 |
|  | L | 5.5 | -30 | -86 | 34 |  |  |  |  |
| Lingual Gyrus | R | 8.9 | 12 | -89 | 4 | 9.0 | 6 | -86 | -2 |
|  | R |  |  |  |  | 8.8 | 14 | -91 | 0 |
|  | R |  |  |  |  | 7.7 | 22 | -90 | -4 |
| Fusiform Gyrus | L | 8.1 | -30 | -37 | -12 | 8.1 | -30 | -38 | -13 |
| Superior Temporal Gyrus | L | 5.7 | -42 | -29 | 7 | 4.5 | -44 | -29 | 5 |
|  | R | 4.1 | 46 | -21 | 8 | 5.0 | 63 | -14 | -1 |
|  | R | 3.8 | 63 | -14 | 1 |  |  |  |  |
| Middle Temporal Gyrus | L | 4.0 | -57 | -8 | -3 | 4.1 | -55 | 3 | -20 |
|  | L |  |  |  |  | 4.0 | -57 | -10 | -5 |
|  | R |  |  |  |  | 4.8 | 53 | -5 | -22 |
| Inferior Temporal Gyrus | L | 4.7 | -57 | -9 | -15 | 7.1 | -59 | -11 | -16 |
|  | R | 5.1 | 53 | -7 | -20 |  |  |  |  |
| Parahippocampal Gyrus | R | 9.8 | 28 | -41 | -8 | 8.6 | 30 | -43 | -10 |
|  | R | 8.7 | 30 | -28 | -20 |  |  |  |  |

Table S3.4: Anatomical regions, peak activation t-values, and Talairach-coordinates for contrast social positive (p) vs. reactive aggressive (a) scenarios in the CON sub-sample (n = 21), separately without (p > a) and with (p > a (cov CFT)) CFT values as covariate; H = hemisphere: L = left, R = right, all statistics p < .001, uncorrected, minimum voxel cluster size k = 10 voxels.

## Supplementary references

Fehr, T. (2012). Neuronale Korrelate der Aggression beim Menschen - virtuelle Medien und reale Lebensumgebung. In: W. Kaminski & M. Lorber (Hrsg.), Gamebased Learning, München, kopaed.

Fehr, T. & Achtziger, A., Roth, G., & Strüber, D. (2014). Neural correlates of the empathic perceptual processing of realistic social interaction scenarios displayed from a first-order perspective. Brain Research, 1583, 141-158, doi:10.1016/j.brainres.2014.04.041.

Fehr, T. & Herrmann, M. (2015). Can modular psychological concepts like affect and emotion be assigned to a distinct subset of regional neural circuits? Comment on "The Quartet Theory of Human Emotions: An Integrative and Neurofunctional model" by S. Koelsch et al. Physics of Life Reviews, 13, 47-49.
